# Distinct gene signatures in aortic tissue from ApoE^-/-^ mice exposed to pathogens or Western diet

**DOI:** 10.1186/1471-2164-15-1176

**Published:** 2014-12-24

**Authors:** Carolyn D Kramer, Ellen O Weinberg, Adam C Gower, Xianbao He, Samrawit Mekasha, Connie Slocum, Lea M Beaulieu, Lee Wetzler, Yuriy Alekseyev, Frank C Gibson, Jane E Freedman, Robin R Ingalls, Caroline A Genco

**Affiliations:** Department of Medicine, Section of Infectious Diseases, Boston University School of Medicine, Boston, MA USA; Boston Medical Center, Boston, MA USA; Clinical and Translational Science Institute, Boston University, Boston, MA USA; Department of Medicine, Division of Cardiovascular Medicine, University of Massachusetts Medical School, Worcester, MA USA; Department of Pathology and Laboratory Medicine, Boston University School of Medicine, Boston University, Boston, MA USA; Department of Microbiology, Boston University School of Medicine, Boston, MA USA

**Keywords:** ApoE^-/-^ mice, *Porphyromonas gingivalis*, *Chlamydia pneumoniae*, Western diet, Gene expression profiling, GSEA, Atherosclerosis, Vascular inflammation, Vulnerable plaque, PPAR

## Abstract

**Background:**

Atherosclerosis is a progressive disease characterized by inflammation and accumulation of lipids in vascular tissue. *Porphyromonas gingivalis* (*Pg*) and *Chlamydia pneumoniae* (*Cp*) are associated with inflammatory atherosclerosis in humans. Similar to endogenous mediators arising from excessive dietary lipids, these Gram-negative pathogens are pro-atherogenic in animal models, although the specific inflammatory/atherogenic pathways induced by these stimuli are not well defined. In this study, we identified gene expression profiles that characterize *P. gingivalis*, *C. pneumoniae,* and Western diet (WD) at acute and chronic time points in aortas of Apolipoprotein E (ApoE^-/-^) mice.

**Results:**

At the chronic time point, we observed that *P. gingivalis* was associated with a high number of unique differentially expressed genes compared to *C. pneumoniae* or WD*.* For the top 500 differentially expressed genes unique to each group, we observed a high percentage (76%) that exhibited decreased expression in *P. gingivalis-*treated mice in contrast to a high percentage (96%) that exhibited increased expression in WD mice. *C. pneumoniae* treatment resulted in approximately equal numbers of genes that exhibited increased and decreased expression. Gene Set Enrichment Analysis (GSEA) revealed distinct stimuli-associated phenotypes, including decreased expression of mitochondrion, glucose metabolism, and PPAR pathways in response to *P. gingivalis* but increased expression of mitochondrion, lipid metabolism, carbohydrate and amino acid metabolism, and PPAR pathways in response to *C. pneumoniae*; WD was associated with increased expression of immune and inflammatory pathways. DAVID analysis of gene clusters identified by two-way ANOVA at acute and chronic time points revealed a set of core genes that exhibited altered expression during the natural progression of atherosclerosis in ApoE^-/-^ mice; these changes were enhanced in *P. gingivalis*-treated mice but attenuated in *C. pneumoniae-*treated mice. Notable differences in the expression of genes associated with unstable plaques were also observed among the three pro-atherogenic stimuli.

**Conclusions:**

Despite the common outcome of *P. gingivalis*, *C. pneumoniae*, and WD on the induction of vascular inflammation and atherosclerosis, distinct gene signatures and pathways unique to each pro-atherogenic stimulus were identified. Our results suggest that pathogen exposure results in dysregulated cellular responses that may impact plaque progression and regression pathways.

**Electronic supplementary material:**

The online version of this article (doi:10.1186/1471-2164-15-1176) contains supplementary material, which is available to authorized users.

## Background

Atherosclerosis is a chronic disease characterized by endothelial dysfunction and inflammation [1–3]. In addition to the significant monetary burden, atherosclerotic vascular disease undermines functional capacity, leads to a greater dependence on hospitalizations and long-term care, and is a risk factor for the development of myocardial infarction and heart failure [4]. A greater understanding of mechanisms and mediators of vascular dysfunction and inflammatory processes in the aortic vasculature that manifest as atherosclerotic disease are needed in order to promote the development of novel prevention and treatment strategies.

Clinical studies have shown that atherosclerotic plaque in the aortic arch and innominate arteries is commonly observed in patients at risk for stroke, myocardial infarction, atrial fibrillation, and peripheral artery disease and that plaque in the aorta is an indication of generalized atherosclerosis [2]. While retention of lipoprotein into the sub-endothelial vascular layer is believed to be the initiating stimulus leading to the development of atherosclerosis, activation of multiple pathways related to vascular inflammation and dysfunction sustain the process by stimulating recruitment of leukocytes and immune cells into the sub-endothelial layer [1]. Differentiation of monocytes into tissue resident macrophages that engulf and oxidize lipids to become inflammatory foam cells is also a hallmark of atherosclerosis [1].

One of the well-defined risk factors for the development of atherosclerosis is diet-induced obesity, which is on the rise in Western societies [4]. Mounting evidence in humans supports an etiological role for the microbiota in inflammatory atherosclerosis. Recent studies have established that common chronic infections may account for up to 40% of newly developed atherosclerosis independent of genetic risk factors [5]. The Gram-negative bacteria, *Porphyromonas gingivalis (P. gingivalis)* and *Chlamydia pneumoniae (C. pneumoniae),* have been associated with the development and acceleration of plaque burden in humans and these observations have been validated in animal models [1, 6–19]. Both of these pathogens have a high prevalence of infection in the general population. *P. gingivalis* is an oral pathogen strongly associated with periodontal disease, one of the most common chronic diseases with a prevalence between 10-60% of adults [20]. *P. gingivalis* promotes chronic systemic inflammation by disrupting host immune responses and altering the composition of microbial communities [21–24] *. C. pneumoniae* is an obligate intracellular bacterium that infects the respiratory tract and is a major cause of pneumonia in humans. An estimated 2-5 million cases of pneumonia each year in the United States are attributed to *C. pneumoniae* infection [25]. Approximately 50% of adults have evidence of past infections by age 20 and re-infection throughout life is common [25].

Although extensive research has shown endogenous mediators arising from excessive dietary lipids and the pathogens *P. gingivalis* and *C. pneumoniae* are pro-atherogenic [5, 8, 9, 12, 13], the specific inflammatory/atherogenic pathways induced by these individual stimuli in plaque progression are not well defined. In this study, we examined how exposure to two pathogens associated with atherosclerosis induces modulation of gene expression in aortic tissues using ApoE^-/-^ mice that spontaneously develop atherosclerosis in the absence of an additional pro-atherogenic stimulus. ApoE^-/-^ mice are the most widely used mouse model for the development of atherosclerosis in the absence of additional stimuli and are characterized by increased total plasma cholesterol levels [1, 26–28]. Furthermore, we compared pathogen-induced gene signatures to ApoE^-/-^ mice fed a Western diet. Comparison of gene expression profiles obtained from pathogen treated mice at the acute and chronic time points was also examined to define how these pathogens modulate gene expression during the natural progression of atherosclerosis in ApoE^-/-^ mice.

## Results

### PCA and qRT-PCR analysis

Principal Component Analysis (PCA, Additional file 1: Figure S1) performed using all genes across all samples showed that there was strong separation between chronic and acute *P. gingivalis* treatment samples (dark and light orange) along the PC1 axis, indicating that there was strong time-dependent differential gene expression in this treatment group. Mean fold changes (relative to chronic control) obtained by Taqman real time RT-PCR analysis for 10 genes were in good agreement with microarray results (Additional file 2: Table S1 and Additional file 3: Figure S2).

### Chronic treatment with *P. gingivalis*, *C. pneumoniae,*and WD induces distinct gene expression patterns in aortic tissue

**Figure 1:**
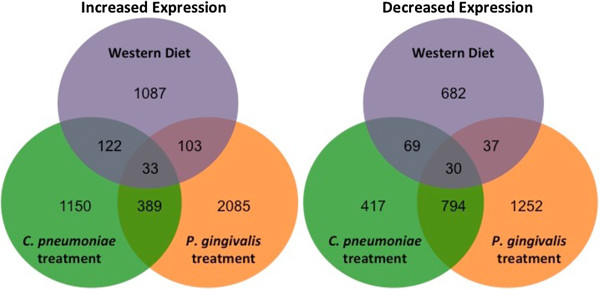
**Chronic time point Venn diagrams.** Venn diagrams depicting the number of genes with significantly increased (left) or significantly decreased (right) expression vs. the chronic control group for each chronic treatment group. The sets of differentially expressed genes result from differential expression analyses for each chronic treatment group vs. the chronic control group using the R environment for statistical computing (version 2.15.1) with a cut off of FDR *q* < 0.25 as described in Methods.

Genes with significant differential expression (FDR *q* < 0.25) in response to the three pro-atherogenic stimuli are tabulated in Figure 1. *P. gingivalis* treatment resulted in a 2-3 times greater number of unique differentially expressed genes compared to the other two treatments. The number of differentially expressed genes common to the two pathogen-treated groups was also substantially larger than the number common to either pathogen-treated group and the WD group.

### Identification of pathways altered by chronic treatment with *P. gingivalis*, *C. pneumoniae,*or WD

To provide insight into the biological pathways and processes that are altered in response to each treatment, we used Gene Set Enrichment Analysis (GSEA) [29–31] to determine whether the members of gene sets involved in curated pathways and processes (as obtained from the Molecular Signatures Database, MSigDB) were nonrandomly distributed across all genes interrogated by the array with respect to each treatment.

The gene sets that were coordinately upregulated (positively enriched) in response to *P. gingivalis* were related to proliferative signaling, matrix remodeling, execution of apoptosis, PDGF signaling, the secretin G-protein receptor family, and the lysosome (Table 1 and Additional file 4: Table S2). The gene sets with the strongest coordinate downregulation (negative enrichment) with *P. gingivalis* treatment were involved in mitochondrial function and glucose metabolism (Table 2 and Additional file 4: Table S3), suggesting that *P. gingivalis* treatment may lead to mitochondrial dysfunction and metabolic imbalance in the aorta. *C. pneumoniae* treatment was associated with the coordinate upregulation of genes involved in redox signaling, lipid metabolism, carbohydrate and amino acid metabolism, the mitochondrion, and vitamin metabolic processes (Table 3 and Additional file 4: Table S4), and the downregulation of genes related to muscle contraction and differentiation and channel activity (Table 4 and Additional file 4: Table S5). These findings suggest that *C. pneumoniae* treatment alters the redox state and metabolism of lipids that may promote the dedifferentiation of smooth muscle cells in aortic tissue. The majority of gene sets that were coordinately upregulated in response to WD were those involved in defense and immune function; others included pathways related to macromolecular degradation and cell cycle regulation (Table 5 and Additional file 4: Table S6). Gene sets that were negatively enriched in response to WD included tight junction regulation, receptor signaling, muscle proteins and channel activity (Table 6 and Additional file 4: Table S7). These findings suggest that dietary lipids and cholesterol are sensed by the innate and adaptive immune system in aortic tissue in a manner that promotes inflammation and loss of vascular barrier function.

**Table 1:** **Gene set enrichment analysis**

Rank	Gene set
**1**	**Basal cell carcinoma**
**3**	**Nucleachromosome part**
**5**	**Regulation of gene expression epigenetic**
**6**	**KEGG melanogenesis**
**8**	**DNA replication**
2	DNA dependent DNA replication
11	Regulation of DNA replication
**9**	**Proteinaceous extracellular matrix**
4	Extracellular matrix part
7	Basement membrane
13	Extracellular matrix
**10**	**KEGG hedgehog signaling pathway**
**12**	**Reactome cell extracellular matrix interactions**
**14**	**Reactome E2F mediated regulation of DNA replication**
**15**	**Reactome apoptotic execution phase**
**16**	**Collagen**
**17**	**Reactome inactivation of APC via direct inhibition of the AP complex**
**19**	**Reactome smooth muscle contraction**
**20**	**Reactome conversion from APC CDC20 to APC CD1 in late anaphase**
**21**	**Reactome signaling by PDGF**
**22**	**KEGG lysosome**
**23**	**Microtubule cytoskeleton**
**24**	**Cell division**
18	Cytokinesis
**25**	**Reactome class B2 secretin family receptors**

Positive enrichment: chronic *P. gingivalis*-treated group vs. chronic control group.

The top 25 *P. gingivalis* gene sets whose member genes are predominantly upregulated with respect to the chronic control group. Column 1 indicates the rank of the gene set based on Normalized Enrichment Score (NES). Column 2 lists the gene set name (bold) as provided by MSigDB. Gene set names and ranks that are not in bold are gene sets that are redundant or partially redundant and included within larger bolded gene sets. The NES ranged from 2.32 (for Basal Cell Carcinoma, the top ranked gene set) to 1.66 (REACTOME Class B2 Secretin Family Receptors, the 25th-ranked gene set). FDR *q* range: 0.001-0.186.

**Table 2:** **Gene set enrichment analysis**

Rank	Gene set
**2**	**Reactome integration of energy metabolism**
**4**	**KEGG oxidative phosphorylation**
**5**	**KEGG parkinsons disease**
**7**	**Mitochondrial part**
**8**	**Mitochondrian**
10	Mitochondrial membrane
11	Mitochondrial envelope
17	Mitochondrial membrane part
**9**	**KEGG citrate cycle tca cycle**
**12**	**KEGG alzheimers disease**
**13**	**Organelle inner membrane**
16	Mitochondrial inner membrane
**14**	**KEGG huntingtons disease**
**15**	**Reactome pyruvate metabolism and TCA cycle**
18	Reactome citric acid cycle
**19**	**KEGG cardiac muscle contraction**
**20**	**Reactome diabetes pathways**
1	Reactome glucose regulation of insulin secretion
3	Reactome electron transport chain
6	Reactome regulation of insulin secretion
**21**	**Reactome glucose metabolism**
**22**	**Energy derivation by oxidation of organic compounds**
23	Cellular respiration
**24**	**Regulation of heart contraction**
**25**	**KEGG PPAR signaling pathway**

Negative enrichment: chronic *P. gingivalis*-treated group vs. chronic control group.

The top 25 *P. gingivalis* gene sets whose member genes are predominantly downregulated with respect to the chronic control group. Column 1 indicates the rank of the gene set based on Normalized Enrichment Score (NES). Column 2 lists the gene set name (bold) as provided by MSigDB. Gene set names and ranks that are not in bold are gene sets that are redundant or partially redundant and included within larger bolded gene sets. The NES ranged from -3.24 (for REACTOME Integration of Energy Metabolism, the top ranked gene set) to -2.33 (KEGG PPAR Signaling Pathway, the 25th-ranked gene set). FDR *q* < 1 × 10^-30^.

**Table 3:** **Gene set enrichment analysis**

Rank	Gene set
**1**	**Microbody**
**2**	**Peroxisome**
20	Peroxisome organization and biogenesis
7	KEGG propanoate metabolism
**3**	**KEGG peroxisome**
12	Reactome peroxisomal lipid metabolism
**4**	**Kegg PPAR signaling pathway**
**5**	**Reactome metabolism of lipids and lipoproteins**
6	Reactome regulation of lipid metabolism by peroxisome proliferator activated receptor alpha
22	Reactome cholesterol biosynthesis
23	Reactome synthesis of bile acids and bile salts via 7 alpha hydroxycholesterol
**8**	**KEGG valine leucine and isoleucine degradation**
**7**	Kegg propanoate metabolism
**9**	**KEGG fatty acid metabolism**
**10**	**Reactome metabolism of vitamins and cofactors**
**11**	**KEGG pyruvate metabolism**
7	KEGG propanoate metabolism
**13**	**KEGG glycerolipid metabolism**
**15**	**Lipid catabolic process**
24	Cellular lipid catabolic process
**16**	**Mitochondrion**
14	Mitochondrial lumen
17	Mitochondrial matrix
7	KEGG propanoate metabolism
**18**	**Reactome branched chain amino acid catabolism**
**19**	**Cofactor metabolic process**
**21**	**KEGG biosynthesis of unsaturated fatty acids**
**25**	**Vitamin metabolic process**

Positive enrichment: chronic *C. pneumoniae*-treated group vs. chronic control group.

The top 25 *C. pneumoniae* gene sets whose member genes are predominantly upregulated with respect to the chronic control group. Column 1 indicates the rank of the gene set based on Normalized Enrichment Score (NES). Column 2 lists the gene set name (bold) as provided by MSigDB. Gene set names and ranks that are not in bold are gene sets that are redundant or partially redundant and included within larger bolded gene sets. The NES ranged from 2.64 (for Microbody Peroxisome, the top ranked gene set) to 2.03 (Vitamin Metabolic Process, the 25th-ranked gene set). FDR *q* range: 0-0.001.

**Table 4:** **Gene set enrichment analysis**

Rank	Gene set
**1**	**KEGG hypertrophic cardiomyopathy**
**2**	**KEGG dilated cardiomyopathy**
**3**	**Reactome muscle contraction**
4	Reactome striated muscle contraction
**5**	**Muscle development**
21	Skeletal muscle development
**6**	**Actin cytoskeleton**
**8**	**Structural molecule activity**
7	Structural constituent of muscle
**9**	**Regulation of multicellular organismal process**
12	Regulation of heart contraction
25	Regulation of muscle contraction
**10**	**KEGG cardiac muscle contraction**
**11**	**Cytoskeletal protein binding**
23	Actin binding
**13**	**KEGG arrhythmogenic right ventricular cardiomyopathy ARVC**
**14**	**Contractile fiber**
**15**	**Contractile fiber part**
19	Myofibril
**16**	**Heart development**
**20**	**Gated channel activity**
17	Voltage gated channel activity
18	Voltage gated cation channel activity
**22**	**Cation channel activity**
**24**	**Cytoskeletal part**

Negative enrichment: chronic *C. pneumoniae*-treated group vs. chronic control group.

The top 25 *C. pneumoniae* gene sets whose member genes are predominantly downregulated with respect to the chronic control group. Column 1 indicates the rank of the gene set based on Normalized Enrichment Score (NES). Column 2 lists the gene set name (bold) as provided by MSigDB. Gene set names and ranks that are not in bold are gene sets that are redundant or partially redundant and included within larger bolded gene sets. The NES ranged from -2.79 (for KEGG Hypertrophic Cardiomyopathy, the top ranked gene set) to -2.23 (Regulation of Muscle Contraction, the 25th-ranked gene set). FDR *q* < 1 × 10^-30^.

**Table 5:** **Gene set enrichment analysis**

Rank	Gene set
**1**	**KEGG lysosome**
**2**	**Reactome signaling in immune system**
10	Reactome innate immunity signaling
24	Cell surface interactions at the vascular wall
**3**	**KEGG natural killer cell mediated cytotoxicity**
**4**	**KEGG systemic lupus erythematosus**
**6**	**KEGG B cell receptor signaling pathway**
**7**	**Leishmania infection**
**8**	**KEGG toll like receptor signaling pathway**
**9**	**Immune system process**
5	Immune response
**11**	**Reactome toll receptor cascades**
**13**	**Reactome S phase**
15	Reactome synthesis of DNA
**14**	**KEGG FC gamma R mediated phagocytosis**
**16**	**Reactome host interactions of HIV factors**
**17**	**Reactome M G1 transition**
12	DNA replication pre initiation
**18**	**KEGG T cell receptor signaling pathway**
**19**	**Defense response**
**20**	**KEGG nod like receptor signaling pathway**
**21**	**KEGG hematopoietic cell lineage**
**22**	**Reactome G1 S transition**
12	DNA replication pre initiation
**23**	**KEGG chemokine signaling pathway**
**25**	**Reactome cell cycle checkpoints**

Positive enrichment: Western diet group vs. chronic control group.

The top 25 Western diet gene sets whose member genes are predominantly upregulated with respect to the chronic control group. Column 1 indicates the rank of the gene set based on Normalized Enrichment Score (NES). Column 2 lists the gene set name (bold) as provided by MSigDB. Gene set names and ranks that are not in bold are gene sets that are redundant or partially redundant and included within larger bolded gene sets. The NES ranged from 2.97 (for KEGG Lysosome, the top ranked gene set) to 2.42 (REACTOME Cell Cycle Checkpoints, the 25th-ranked gene set). FDR *q* < 1 × 10^-30^.

**Table 6:** **Gene set enrichment analysis**

Rank	Gene set
**1**	**G protein coupled receptor activity**
6	Rhodopsin like receptor activity
14	Peptide receptor activity
**2**	**Apical junction complex**
**3**	**Apicolateral plasma membrane**
**4**	**Neurotransmitter binding**
5	Neuropeptide receptor activity
7	Neurotransmitter receptor activity
10	Neuropeptide binding
**9**	**KEGG neuroactive ligand receptor interaction**
**11**	**Reactome amine ligand binding receptors**
24	Amine receptor activity
**12**	**Intercellular junction**
8	Tight junction
**13**	**Structural constituent of muscle**
**15**	**Calcium channel activity**
**16**	**Feeding behavior**
**18**	**Voltage gated channel activity**
17	Voltage gated potassium channel activity
20	Voltage gated cation channel activity
**19**	**KEGG basal cell carcinoma**
**21**	**Contractile fiber**
**22**	**Anion transmembrane transporter activity**
**23**	**Reactome tight junction interactions**
**25**	**Digestion**

Negative enrichment: Western diet group vs. chronic control group.

The top 25 Western diet gene sets whose member genes are predominantly downregulated with respect to the chronic control group. Column 1 indicates the rank of the gene set based on Normalized Enrichment Score (NES). Column 2 lists the gene set name (bold) as provided by MSigDB. Gene set names and ranks that are not in bold are gene sets that are redundant or partially redundant and included within larger bolded gene sets. The NES ranged from -3.43 (for G Protein Coupled Receptor Activity, the top ranked gene set) to -1.92 (Digestion, the 25th-ranked gene set). FDR q range: 0 – 0.015.

### Functional classification of clusters of genes differentially expressed with respect to chronic atherogenic stimuli

**Figure 2:**
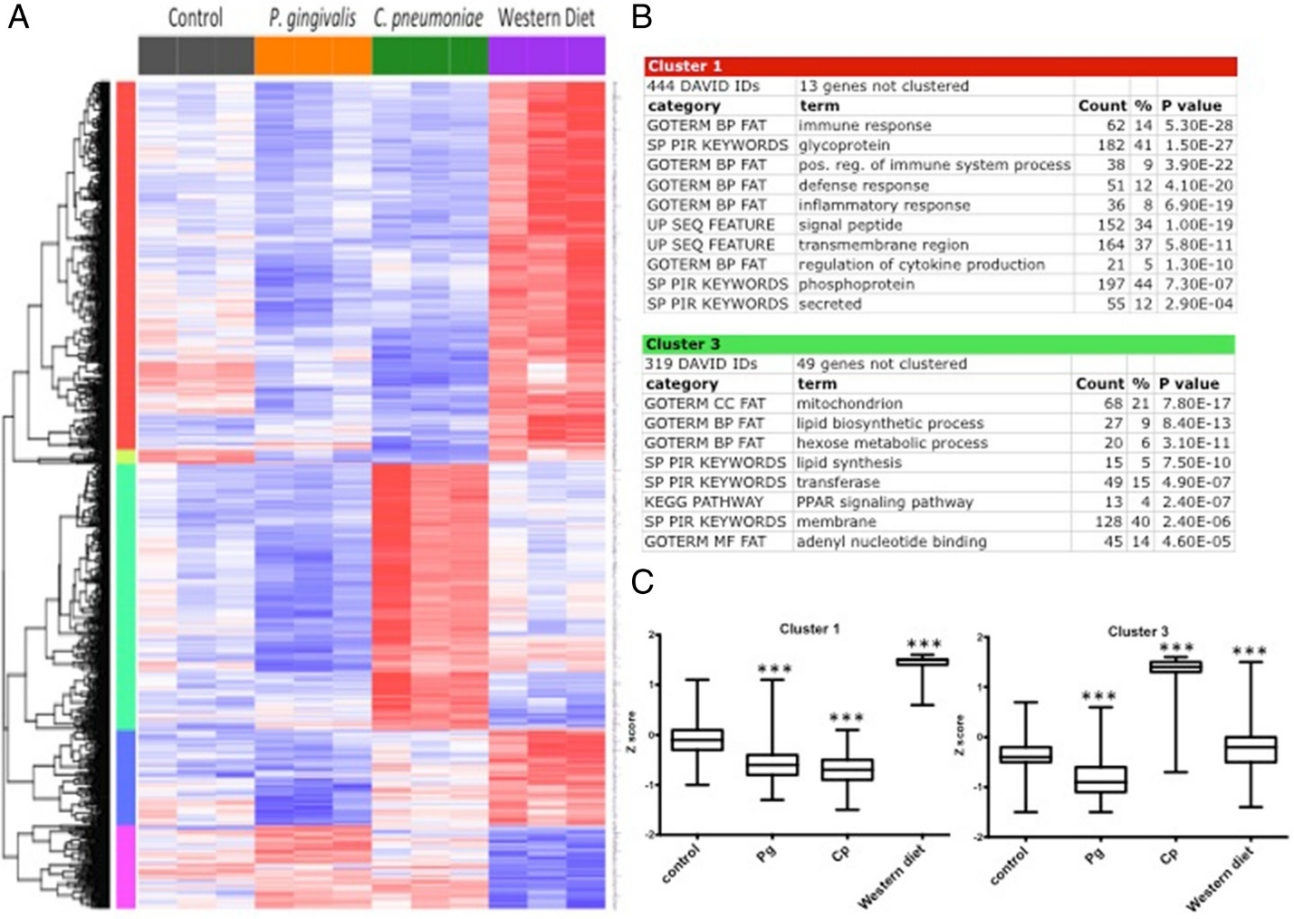
**Chronic time point cluster analysis.** The top 1000 differentially expressed genes at the chronic time point with 5 distinct clusters. **A**. Heat map shows relative expression among all groups. Clusters are color-coded by row sidebars: red (cluster 1), chartreuse (cluster 2), mint green (cluster 3), blue (cluster 4), and magenta (cluster 5); and dendrogram is left of the color-coded sidebars. Each row corresponds to a gene (gene symbols are listed to the right of each row) and each column to a sample. The colors are scaled by row; red and blue indicate 2 standard deviations above or below the mean (white), respectively. At the arbitrary cutoff of 1000 genes, the chronic time point one-way ANOVA FDR *q* value was < 0.019 and the *p* value was < 8.73 × 10^-4^. **B**. DAVID analysis of clusters 1 and 3. Gene enrichment is indicated by *p* values (EASE scores, a modified Fisher exact *p* value). **C**. Box and whisker plots of the mean expression (log2) for Clusters 1 and 3 reflect patterns seen on heat map. *** = *p* < 0.0001 chronic treatment group vs. chronic control group by Mann-Whitney test.

The 1000 genes with the greatest significance by one-way ANOVA across the four chronic treatment groups (control, *P. gingivalis*, *C. pneumoniae*, and WD) were assigned to several groups using hierarchical clustering (Figure 2A-C). At the arbitrary cutoff of 1000 genes, the chronic time point ANOVA FDR *q* value was < 0.019 and the *p* value was < 8.73 × 10^-4^. The DAVID functional classification tool was used to extract biological meaning from each of the clusters (Figure 2B). The three pro-atherogenic stimuli produced strikingly different patterns of differential gene expression. Genes involved in immunity and inflammation (Cluster 1) were coordinately upregulated in response to WD, but were largely unchanged in response to either pathogen. By contrast, genes involved in lipid synthesis and PPAR signaling (Cluster 3) were markedly increased in response to *C. pneumoniae* treatment but were unchanged in the other groups. Finally, two smaller clusters suggest that the Hedgehog pathway is suppressed in WD mice (Cluster 5) and that treatment with either pathogen decreased the expression of contractile proteins (Cluster 2); however, these results were based on a small number of genes and should be interpreted with caution (Figure 2A and Additional file 5: Figure S3A and B).

### Time-dependent changes in aortic gene expression in mice treated with *P. gingivalis*or *C. pneumoniae*

**Figure 3:**
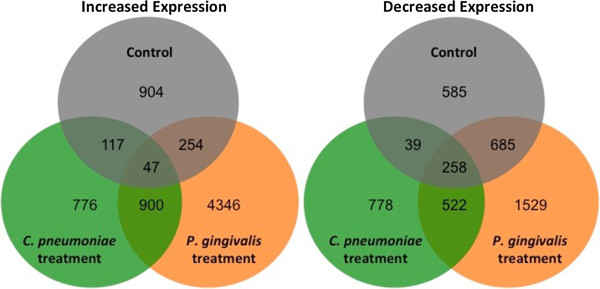
**Acute to chronic time point Venn diagrams.** Venn diagrams depicting the number of genes with significantly increased (left) or significantly decreased (right) expression vs. the acute group for each chronic group. The sets of differentially expressed genes result from differential expression analyses for each acute group vs. its corresponding chronic group using the R environment for statistical computing (version 2.15.1) with a cut off of *p* < 0.05 as described in Methods.

To examine the acute response in aortic tissue following pathogen exposure, RNA samples were obtained one day after the last treatment with *P. gingivalis* or *C. pneumoniae* (see Methods). Genes with nominally significant differential expression (*p* < 0.05) between the chronic and acute time points in untreated or *P. gingivalis*- or *C. pneumoniae*-treated ApoE^-/-^ mice are tabulated in Figure 3. Differential expression in all three groups was greater than expected by chance, and as in the comparison between groups at the chronic time point alone (Figure 1), *P. gingivalis* treatment produced the largest amount of differential gene expression, and the genes regulated by the two pathogens overlapped substantially. For the top 500 differentially expressed genes observed at the acute to chronic time points, aortic tissue from ApoE^-/-^ mice fed a normal chow diet was characterized by a balance of genes with increased and decreased expression; *P. gingivalis* treatment was skewed towards decreased expression (see Additional file 6: GeneLists). Because time-dependent differential expression was less robust in the *C. pneumoniae*-treated group, these changes may reflect inhibition of natural changes that occur with time in the aortic tissue from ApoE^-/-^ mice fed a normal chow diet.

### Functional classification of genes with varying kinetics of response to *P. gingivalis*or *C. pneumoniae*treatment

**Figure 4:**
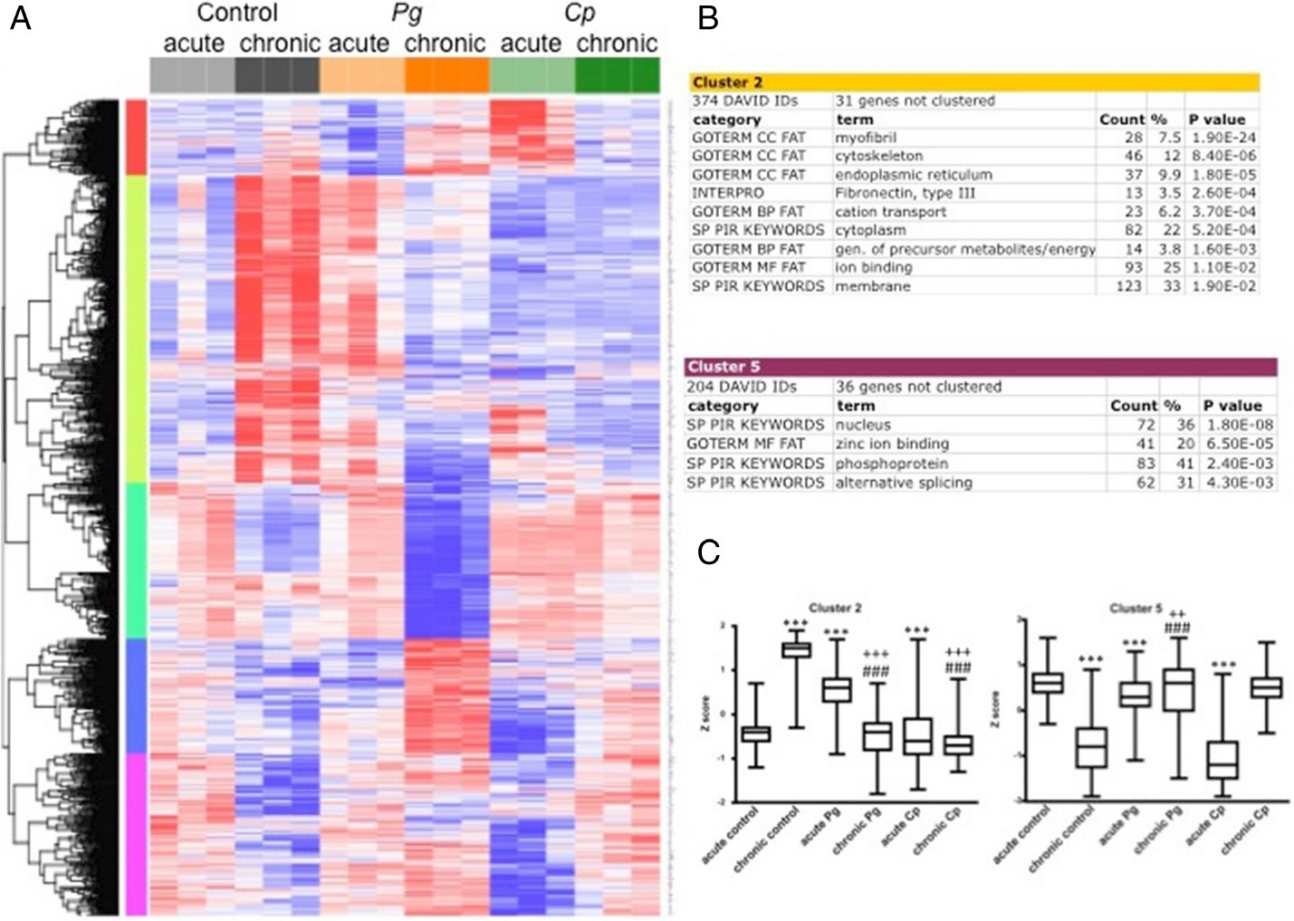
**Acute to chronic time point cluster analysis.** The top 1000 differentially expressed genes for acute and chronic time points: the effect of group, time, and group x time interactions determined by two-way ANOVA are grouped into 5 distinct clusters. **A**. Heat map depicts relative expression among all groups and time points. Clusters are color-coded by row sidebars: red (cluster 1), chartreuse (cluster 2), mint green (cluster 3), blue (cluster 4), and magenta (cluster 5); and dendrogram is left of the color-coded sidebars. Each row corresponds to a gene (gene symbols are listed to the right of each row) and each column to a sample. The colors are scaled by row; red and blue indicate 2 standard deviations above or below the mean (white), respectively. At the arbitrary cutoff of 1000 genes, the two-way ANOVA interaction (group:timepoint) FDR *q* value was < 0.117 and the *p* value was < 5.52 × 10^-3^. **B**. DAVID analysis of clusters 2 and 5. Gene enrichment is indicated by *p* values (EASE scores, a modified Fisher exact *p* value). **C**. Box and whisker plots of the mean expression (log2) for Clusters 2 and 5 reflect patterns seen on heat map. ***p < 0.0001 vs. acute control; ###*p* < 0.0001 vs. chronic control; +++*p* < 0.0001 vs. acute treatment; ++*p* < 0.001 vs. acute treatment by Mann-Whitney test.

Using two-factor ANOVA, we then analyzed the capacity of either pathogen to alter normal time-dependent changes in gene expression that occurred in ApoE^-/-^ mice. A clustering heat map of the 1000 genes with the strongest interaction effect between time and treatment is shown in Figure 4. At the arbitrary cutoff of 1000 genes, the ANOVA interaction (group:timepoint) FDR *q* value was < 0.117 and the *p* value was < 5.52 × 10^-3^. The expression of genes encoding myofibril, cytoskeletal, and ion binding/transport proteins (cluster 2) greatly increased over time in untreated ApoE^-/-^ mice. However, treatment with either pathogen prevented or reversed this effect: the expression of these genes was increased and subsequently downregulated in the acute or chronic *P. gingivalis*-treated groups, respectively, and was unchanged in either *C. pneumoniae* treatment group. Conversely, the expression of a number of zinc-finger transcription factors (cluster 5) was downregulated over time in untreated mice, but was decreased and then subsequently upregulated in the acute or chronic *C. pneumoniae*-treated groups, respectively, and was unchanged in either *P. gingivalis* treatment group. A third noteworthy pattern was defined by a group of genes with functions in fatty acid metabolism and PPAR signaling whose expression was moderately downregulated over time in untreated animals (cluster 3); this downregulation was greatly amplified in mice treated with *P. gingivalis* but was completely abrogated in mice treated with *C. pneumoniae* (Additional file 7: Figure S4).

### Functional classification of clusters of genes differentially expressed with respect to acute atherogenic stimuli

As before, the 1000 genes with the greatest significance by one-way ANOVA across the three acute treatment groups (control, *P. gingivalis*, and *C. pneumoniae*) were assigned to several groups using hierarchical clustering (Additional file 8: Figure S5-1A). Acute treatment with *P. gingivalis* produced little differential gene expression, whereas acute treatment with *C. pneumoniae* resulted in large changes in gene expression. Genes whose expression increased with *C. pneumoniae* treatment (cluster 1) represented G-protein coupled signaling, viral myocarditis, antigen processing and presentation, and membrane genes. By contrast, nearly half of the genes whose expression decreased with *C. pneumoniae* treatment (cluster 5) encoded phosphoproteins, with the remainder encoding proteins involved in alternative splicing, the endoplasmic reticulum, and glycoproteins and secreted proteins. The remaining clusters were not remarkable due to lack of annotation to a DAVID pathway or of marginal significance (Additional file 8: Figure S5-1B). The three remaining clusters were not noteworthy (Additional file 9: Figure S5-2).

### Analysis of genes associated with unstable plaque

**Figure 5:**
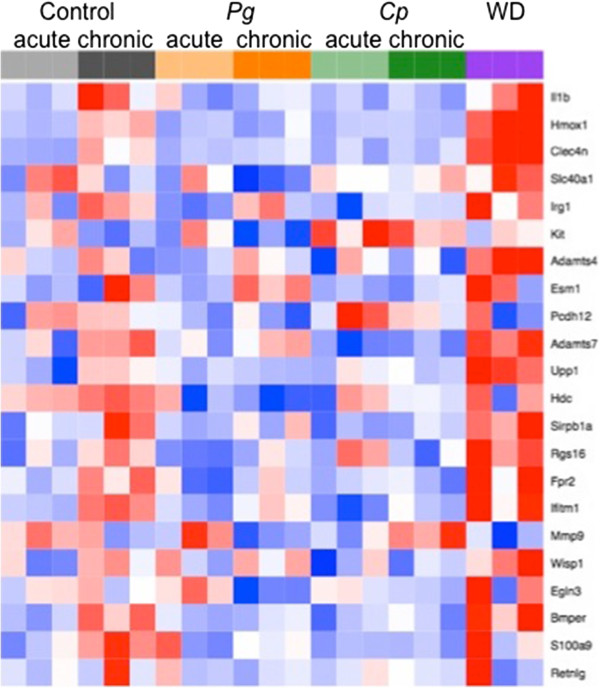
**Genes associated with unstable plaque.** Heat map showing expression of genes associated with unstable plaque identified in Chen, et al. [32].

A recent study identified 22 genes whose expression in the aortic tissue of ApoE^-/-^ mice was associated with unstable plaque [32]. We observed increased expression of several of these genes in aortic tissue from untreated ApoE^-/-^ mice fed a normal chow diet, which represents the natural progression of atherosclerosis (Figure 5 and Additional file 10: Figure S6); and, in ApoE^-/-^ WD mice, we observed an even greater increase in expression of some of these genes. *P. gingivalis* treatment blunted the increase in expression of some genes and induced the expression of others. Treatment with *C. pneumoniae* prevented the increase in all of the unstable plaque genes with the exception of Mmp9, which was increased over time in the *C. pneumoniae-*treated group but decreased over time in untreated ApoE^-/-^ mice.

## Discussion

In this study, we applied microarray analysis to define gene signatures in aortic tissues obtained from mice fed a Western diet (WD) or in mice treated with the oral pathogen *P. gingivalis* (*P. gingivalis*), or with the pulmonary pathogen *C. pneumoniae* (*C. pneumoniae*). Examination of gene expression profiles at the chronic time point enabled us to identify treatment-specific pro-atherogenic pathways rather than simply the genes expressed in established plaque. Our results in untreated ApoE^-/-^ mice are consistent with a recent microarray study by Papadodima et al. [26] that documented changes in gene expression in aortic tissue of ApoE^-/-^ mice at three time points during the natural progression of atherosclerosis in the absence of additional pro-atherogenic stimuli. We identified common core sets of genes that were either increased or decreased in response to each of these pro-atherogenic stimuli.

Contemporary analysis tools including GSEA and DAVID analysis were then applied. GSEA approach is more powerful than single gene analysis, which merely highlights the top up- or downregulated genes and may overlook effects on specific pathways [29]. Pathway analysis showed a markedly greater number of gene sets that were different among the three pro-atherogenic stimuli, suggesting that underlying vascular inflammation and dysfunction leading to atherosclerosis differ mechanistically and in the functional pathways leading to atherosclerosis progression depending upon stimulus. These findings are significant in that this is the first study that has performed direct, side-by-side, comparison of genome-wide aortic gene expression changes in three well-recognized models of atherosclerosis development.

Treatment with WD, *P. gingivalis* or *C. pneumoniae* has pro-atherogenic effects that include alterations in macrophage function, cholesterol homeostasis, and endothelial dysfunction [13, 33]. Signaling through innate immune Toll-like receptors (TLRs) expressed on immune cells and the endothelium may be a common link among the three stimuli; however, notable differences in TLR usage as well as features unique to each of the two pathogens may account for the differences in signaling pathways activated and repressed. TLR1, TLR2, and TLR4 RNA and protein are expressed at high levels in endothelial cells and macrophages in human atherosclerotic plaque biopsies and the lipid-rich atherosclerotic lesions in the aortic root of mice [34, 35]. A role for TLRs in high-fat-diet-induced atherosclerosis in animal models has been shown in studies using ApoE^-/-^ TLR2^-/-^ and ApoE^-/-^ TLR4^-/-^ mice. Interestingly specificity in TLR2 and TLR4 signaling by *P. gingivalis* and *C. pneumoniae* has been proposed to define specific inflammatory pathways unique to each organism [36–43]. *P. gingivalis* expresses heterogeneous LPS lipid A structures that weakly activate TLR4 but can also act as a TLR4 antagonist [44, 45], resulting in alterations in signaling through TLR4 that promote low-grade, chronic inflammation at distant sites including the aorta [46]. *C. pneumoniae* induces its proinflammatory signaling primarily through TLR2 but also expresses LPS and signals through TLR4 [47]. The unique feature of *C. pneumoniae* is that it gains entry into monocytes as elementary bodies (EBs), which are infectious but metabolically inert. After entering macrophages or monocytes, EBs can rapidly differentiate to a replicative form known as reticulate bodies (RBs), and start bacterial replication [48]. RBs use the host’s metabolic metabolism and may find a favorable environment within a lipid-laden plaque to complete its replication. Infected monocytes may circulate to distant sites to promote vascular inflammation [49]. *C. pneumoniae* may increase adherence of macrophages to endothelial cells through expression of its virulence factor, HSP 60, which has been shown to promote monocyte attachment to endothelium, and to promote extravasation to sub-endothelial layer, where it oxidizes LDL and promotes foam cell formation [50, 51], a hallmark of atherosclerosis.

Most published studies involving animal models of atherosclerosis have used high dietary lipids and cholesterol (various modifications of Western style diets), and these studies have formed the basis for consensus around the mechanisms underlying the development of atherosclerosis. As a result, the use of statin drugs, which target cholesterol and lipid handling, are the most widely used class of drugs in the field of cardiovascular medicine. A recent study in Circulation [3] showed that despite the improvement of risk with optimum statin therapy in patients with cardiovascular disease, many patients demonstrate atheroma progression and additional cardiovascular events, suggesting that additional mechanisms are at play, highlighting the need to identify novel therapeutic strategies that combat the additional cardiovascular risk. Recent research implicates vascular inflammation and endothelial dysfunction as a factor involved in the initiation, progression, and instability of atherosclerotic plaques and elevations of serum inflammatory biomarkers consistently associated with the risk of experiencing a cardiovascular event, providing further evidence for systemic inflammation involvement in atherosclerotic cardiovascular disease [4].

Given the prevalence of diet-induced obesity and infection with *P. gingivalis* and *C. pneumoniae* in the general population and the likelihood of co-morbidity of obesity with chronic or recurring infection with these common pathogens, these findings suggest that the development of atherosclerosis in humans is likely more complex and multifactorial than previously appreciated. Atherosclerotic plaques undergo both progressive and regressive changes, which affect their size and stability; regression in lesion area can increase plaque stability. Plasma cholesterol lowering has been associated with regression, and a recent study identified PPAR-gamma as a “master regulator” of regression in early lesions [52]. Our GSEA analysis showed that *C. pneumoniae* treatment upregulated the PPAR pathway while *P. gingivalis* treatment downregulated this pathway and thus may indicate a role for *C. pneumoniae* in regression and stability of early lesions, while *P. gingivalis* may inhibit plaque regression. *P. gingivalis* decreased pathways involved in mitochondrial function, suggesting that *P. gingivalis* promotes mitochondrial dysfunction, which is associated with cardiovascular risk, vascular dysfunction and plaque development [53]. Western diet group had significant reduction in tight junction pathways and expression of genes encoding claudins, proteins involved in maintenance of cell-cell junctions. This finding is consistent with a recent study that showed that Western diet promotes atherosclerosis in part via loss of tight junction control [54]. A surprising finding from the present study was that immune and inflammatory pathways were markedly upregulated in response to Western diet. It has recently come to light that certain lipids in Western style diets are either endogenous ligands of TLRs or assist with pattern recognition of TLRs [55, 56]. Consistent with this, prior studies have shown that TLR2, TLR4, and MyD88 knockout mice on an ApoE^-/-^ background had decreased atherosclerotic aortic plaque in response to a Western diet [36, 38, 39]. A recent microarray study by Hyvarinen *et al*., compared gene expression in adipose tissue following chronic infection with *C. pneumoniae* and/or a periodontal pathogen, *A. actinomycetemcomitans* (*Aa*) and revealed significant enrichment in inflammation associated pathways by *Aa* or a combination of *Aa* and *C. pneumoniae* but not by *C. pneumoniae* alone as compared to a control group [57].

Of interest was the increased expression of a number of genes associated with vulnerable plaque previously identified by Chen et al. [32] in aortic tissues obtained from mice at the chronic phase compared to tissues obtained at the acute phase. These genes were Bmper, Hdc, Ifitm1, S100a9, Upp1, Adamts7, Fpr2, Clec4n, Hmox1 and Irg1. Our findings demonstrate that expression of these genes increases as part of the natural progression of atherosclerosis in ApoE^-/-^ mice in the absence of an additional pro-atherogenic stimulus. Importantly, the increased expression of these genes was blunted at the chronic time point in mice treated with *C. pneumoniae.* Expression of Kit was increased in the *C. pneumoniae* -treated group compared to the chronic control group. In addition, *P. gingivalis* treatment blunted the increase in expression of Bmper, Hdc, Ifitm1, S100a9, Upp1 and Hmox1. Expression of Egln3 was decreased in the *P. gingivalis*-treated group compared to the chronic control group. Expression of Clec4n and Hmox1 was increased in the Western diet group compared to the chronic control group. The identification of differences in the effects of each of the pro-atherogenic stimuli on expression of important vulnerable plaque genes may result in differences in the nature and composition of the atherosclerotic plaque, which will be explored in future studies.

### Limitations of the study

Several limitations of the study should be noted. We could not simultaneously perform a side-by-side quantification of the degree of the aortic plaque and the pathological features assessed by histology induced by the three atherogenic stimuli. We also could not determine the contribution of possible alterations in gut microbiota induced by each of the pro-atherogenic treatments or following antiobiotics pre-treatment in the *P. gingivalis* group. We could not validate our findings with regard to establishing that the pathways we identified are mechanistically linked to each pro-atherogenic stimulus at the protein level. We also acknowledge the limitations of using mouse models to draw conclusions about human diseases, which are more complex and multifactorial. Future studies will also investigate the effects of comorbidity with 2 or more pro-atherogenic stimuli, which resembles scenarios that occur clinically.

## Conclusions

The present study suggests that *P. gingivalis* treatment may promote atherosclerosis by stimulating mitochondrial dysfunction and inhibiting the egress of endogenous lipids from the vessel wall, whereas *C. pneumoniae* may promote atherosclerosis by enhancing lipid uptake and metabolism into the vessel wall, and WD may promote atherosclerosis by activation of inflammatory and immune pathways that enhance leukocyte adhesion and endothelial dysfunction.

## Methods

### Ethics statement

All experiments were carried out in accordance with the recommendations in the Guide for the Care and Use of Laboratory Animals of the National Institutes of Health and study protocols were approved by the Boston University Institutional Animal Care and Use Committee. All experimental procedures involving pathogenic bacteria were carried out with approval from the Boston University Institutional Biosafety Committee.

### Mouse randomization and groups

Male ApoE^-/-^ mice on C57BL/6 background (Jackson Laboratories) of 8 weeks of age were randomly assigned into a chronic exposure cohort and an acute exposure cohort. The chronic exposure cohort consisted of the following groups: *P. gingivalis* treatment (chronic *P. gingivalis*, N = 3), *C. pneumoniae* treatment (chronic *C. pneumoniae*, N = 3), WD (chronic WD, N = 3), and Control (N = 3). The acute exposure cohort consisted of the following groups: *P. gingivalis* treatment (acute *P. gingivalis*, N = 3), *C. pneumoniae* treatment (acute *C. pneumoniae*, N = 3), and Control (N = 3). Mice were fed a normal chow diet (Global 2018; Harlan Teklad, Madison, WI) with the exception of the WD group. Control mice received no additional treatment after randomization and assignment to groups. Mice in the chronic exposure cohort were euthanized 9 weeks after the last bacterial treatment or after 9 weeks on WD; this represents a time point prior to the development of overt aortic plaque as observed approximately 13 weeks following *P. gingivalis*, *C. pneumoniae*, or WD [8, 11, 12, 46, 58–64]. Mice in the acute cohort were euthanized one day after the last bacterial treatment. At the end of each study period, the aortas were dissected and aortic RNA was isolated and used for microarray analysis to compare the acute and chronic responses of the pro-atherogenic stimuli on transcriptional profiles (For experimental protocol, see Additional file 11: Figure S7).

### Pro-atherogenic treatment

*P. gingivalis* strain 381 was grown anaerobically on blood agar plates (Becton Dickinson) and used to seed-inoculate brain heart infusion broth (pH 7.4; Becton Dickinson) supplemented with yeast extract (Becton Dickinson), hemin (10 μg/ml; Sigma), and menadione (1 μg/ml). CFUs were standardized at an OD of 1 at 660 nm (equivalent to 1 × 10^9^ CFU/ml) by spectrophotometry (ThermoSpectronic Genesys20). The protocol for *P. gingivalis* oral infection of ApoE^-/-^ mice was as previously described by our group and characterized for the acceleration of atherosclerosis progression [8, 10, 58]. Briefly, mice were given antibiotics (Sulfatrim; Hi-Tech Parmacal) *ad libitum* in the drinking water for 10 days followed by 2-day antibiotic-free period. Mice were then given one hundred μl of *P. gingivalis* (1 × 10^9^ CFU) suspended in vehicle (2% carboxymethylcellulose in PBS), topically applied to the buccal surface of the maxillary gingiva 5 times a weeks for 3 weeks [65]. We have previously shown that oral treatment with *P. gingivalis* using this protocol results in increased serum levels of *P. gingivalis*-specific IgG1, IgG2b, IgG2c and IgG3 [8]. *C. pneumoniae* strain AO3, initially isolated from a human atheroma, was provided by Dr. Charlotte Gaydos (Johns Hopkins University, Baltimore, MD). *Cp* were propagated in L929 fibroblasts grown in RPMI 1640 medium supplemented with 10% FBS at 35°C in a 5% CO_2_ environment as described [66, 67]. Following infection of fibroblasts, cells were harvested and disrupted by glass beads or sonication (Sonicator 4000, Misonix Sonicators, Newtown, CT) and *Cp* separated from cell debris by ultracentrifugation through 32% Renografin. Bacterial titers were calculated as inclusion forming units (IFU) per milliliter. *Chlamydia* stocks were negative for *Mycoplasma* contamination [66]. ApoE^-/-^ mice were inoculated as previously described [66] with *C. pneumoniae* via the intranasal route under light anesthesia using a ketamine/xylazine mix (60–100/5–10 mg/kg i.p., respectively). Mice received 20 μl bacterial suspensions in phosphate buffered saline (PBS) containing 2 × 10^6^ IFU gradient purified *Chlamydia*. Mice received intranasal infection once a week for 3 weeks. Others have previously shown that intranasal treatment with *C. pneumoniae* using this protocol results in dissemination of *C. pneumoniae* from the lung to aortic plaques as detected by immunohistochemistry and PCR [63, 64]. The WD ApoE^-/-^ mouse group received Teklad Adjusted Calorie Diet (42% from fat) (Harlan Catalog # 88137; 0.2% cholesterol) *ad libitum*, a diet that accelerates atherosclerosis in ApoE^-/-^ mice [59–62].

### Dissection of aorta and RNA extraction

Following euthanasia, the thoracic aortic with the three major branches (innominate, left common carotid, left subclavian arteries) was removed for RNA extraction as follows. On ice, the thoracic aorta was dissected from extraneous tissue. Immediately following dissection, the aorta was placed in a cryotube and stored at -80°C for RNA extraction and analysis. Total aortic RNA was isolated using TriZol extraction reagent (Invitrogen) following homogenization using TissueLyser II in pre-cooled blocks (Qiagen, Valencia, CA). RNA was further purified using the RNAeasy kit (Qiagen, Valencia, CA). Sample integrity was verified using RNA 6000 Pico Assay RNA chips run in Agilent 2100 Bioanalyzer (Agilent Technologies, Palo Alto, CA). Total RNA (5 ng) was reverse-transcribed using Ovation Pico WTA System V2 (Nugen, San Carlos, California). The obtained SPIA-amplified cDNA was purified using Agencourt RNA clean XP Purification Beads and fragmented (5 ng) and labeled with biotin using the Encore Biotin Module (NuGEN, San Carlos, California). SPIA-amplified cDNA and fragmented cDNA quality controls were performed by running an mRNA Pico assay in the Agilent 2100 Bioanalyzer.

### Microarray analysis

Labeled, fragmented DNA was hybridized to a Mouse Gene 1.0 ST Array (Affymetrix, Santa Clara, CA) for 18 hours in a GeneChip Hybridization oven 640 at 45°C with rotation (60 rpm). The hybridized samples were washed and stained using an Affymetrix fluidics station 450. After staining, microarrays were immediately scanned using an Affymetrix GeneArray Scanner 3000 7G Plus. Raw Affymetrix CEL files were normalized to produce log2-transformed, Entrez Gene-specific expression values using the implementation of the Robust Multiarray Average (RMA) in the *affy* package in the Bioconductor software suite (version 2.12) and an Entrez Gene-specific probeset mapping from the Molecular and Behavioral Neuroscience Institute (Brainarray) at the University of Michigan (version 14.0.0). Array quality was assessed by computing Relative Log Expression (RLE) and Normalized Unscaled Standard Error (NUSE) using the *affyPLM* Bioconductor package (version 1.34.0); all arrays had median RLE and NUSE values less than 0.1 and 1.05, respectively, indicating that they were of sufficient quality. Principal Component Analysis (PCA) was performed using all genes after *z*-normalizing expression values to a mean of zero and a standard deviation of one across all samples.

Differential gene expression between each experimental treatment group and the control group within each time point (acute time point: *P. gingivalis* or *C. pneumoniae* vs. control; chronic time point: *P. gingivalis* or *C. pneumoniae* or WD vs. control) was assessed using the empirical Bayesian (moderated) *t* test from the *limma* package (version 3.14.4). Differential gene expression between chronic and acute time points within each applicable treatment group (control, *P. gingivalis* and *C. pneumoniae*) was assessed as above. Analyses of variance were performed using the *f.pvalue* function in the *sva* package (version 3.4.0). Correction for multiple hypothesis testing was accomplished using the Benjamini-Hochberg false discovery rate (FDR) [68, 69]. Human homologs of mouse genes were identified using HomoloGene (version 65) [PubMed ID 23193264]. All microarray analyses were performed using the R environment for statistical computing (version 2.15.1).

### Taqman validation

Ten genes were selected for validation of microarray expression levels using Taqman Inventoried Assays and Real Time RT-PCR. RNA isolated from the same aortic tissue used for the microarray was converted to cDNA using the High Capacity cDNA Synthesis Kit (Life Technologies). Real-time relative PCR was performed on cDNA using Taqman-validated exon-spanning assays and Gene Expression Master Mix (Invitrogen) on a StepOne System and software (Life Technologies Applied Biosystems). Genes chosen for validation were those with expression levels that were representative of various patterns of expression (increased expression, decreased expression, no change in expression) in each of the treatment groups relative to control. Validated genes and their assay number were peroxisome proliferator-activated receptor *gamma (*PPAR-gamma, Mm01184322_m1), CD5 molecule-like (CD5l, Mm00437567_m1), lipocalin 2 (Lcn2, Mm01324470_m1), hedgehog interacting protein (Hhip, Mm00469580_m1), C-type lectin domain family 3, member A (Clec3a, Mm01240105_m1), chemokine (C-X-C motif) ligand 13 (Cxcl13, Mm00444533_m1), G-protein signaling modulator 2 (Gpsm2, Mm00512842_m1), uncoupling protein 1 (Ucp1, Mm01244861_m1), creatine kinase, mitochondrial 2 (Ckmt2, Mm01285553_m1), Toll-like receptor 13 (Tlr13, Mm01233818_m1). Gene expression was normalized to expression of beta-actin for each sample and expressed as fold change to a control sample. Expression levels of beta-actin were similar in all samples (ANOVA *p* = 0.65, NS).

### Gene set enrichment analysis (GSEA)

GSEA was used to identify biological terms, pathways, and processes that were overrepresented among the genes that were up- or down-regulated with respect to various pairwise comparisons. A list of all Entrez Gene identifiers (Entrez Gene IDs) interrogated by the array was ranked according to the moderated *t* statistic, and this list was then used to perform a pre-ranked GSEA analysis using the publicly available Molecular Signatures Database (MSigDB, version 3.0, http://www.broadinstitute.org/gsea/msigdb/). Gene sets corresponding to 1625 biologically defined gene sets representing pathways, locations, or functions were derived from the following public databases: Kyoto Encyclopedia of Genes and Genomes (KEGG), Gene Ontology (GO), Biocarta, and Reactome [29–31, 70]. To control for multiple comparisons in GSEA analysis, Benjamini-Hochberg False Discovery Rate (FDR) correction (FDR *q*) was applied [68, 69].

### DAVID annotation

The Database for Annotation, Visualization and Integrated Discovery (DAVID) v6.7 gene annotation tool was used to understand biological meaning for gene lists identified by clusters and GSEA [71, 72].

### Statistical analysis

Statistical analyses for non-microarray data (Taqman validation, box and whisker plots, and analysis of vulnerable plaque genes) were performed using GraphPad Prism 5.0 software. Comparisons among groups were performed using ANOVA followed by a 2-tailed unpaired Student’s t-test or Mann-Whitney U test.

### Availability of supporting data

The data set supporting the results of this article [73] is available in the Gene Expression Omnibus (GEO) repository, the Series record ID is GSE60086 and the private link is: http://www.ncbi.nlm.nih.gov/geo/query/acc.cgi?token=mvchagcyhfgtrit&acc=GSE60086.

## Authors’ information

CAG is the PI of a P01 grant entitled “Role of innate immune system in pathogen induced chronic inflammation” (NIH NIAID AI078894), with FCG, JEF, RRI, CAG as PIs of individual projects. YA is the director of the BU Microarray Core Resource facility, and ACG is a bioinformatics scientist in the BU Clinical and Translational Science Institute (CTSI).

## Electronic supplementary material

Additional file 1: Figure S1: Principal Component Analysis (PCA). Graph showing variance in global expression in each individual sample array in relation to all 21 arrays. The first principal component (PC1, x axis) had a variance of 25% and the second principal component (PC2, y axis) had a variance of 19%. Note that the 3 replicates in each group cluster near each other. Acute control group: light grey 1, 2, 3; chronic control group: dark grey 1, 2, 3; acute *P. gingivalis*-treated group: light orange p1, p2, p3; chronic *P. gingivalis*-treated group: dark orange P1, P2, P3; acute *C. pneumoniae*-treated group: light green c1, c2, c3; chronic *C. pneumoniae*-treated group: dark green C1, C2, C3; WD group: purple W1, W2, W3. The group with the largest variance among the three replicates is the acute *C. pneumoniae*-treated group. (JPEG 27 KB)

Additional file 2: Table S1: Comparison of microarray and RT-PCR expression results for 10 genes. Comparison of mean fold changes in gene expression obtained by microarray analysis and real time RT-PCR for 10 genes at the chronic time point. *P. gingivalis* = *P. gingivalis*-treated group; *C. pneumoniae* = *C. pneumoniae*-treated group. (JPEG 45 KB)

Additional file 3: Figure S2: Taqman validation of 10 genes. One-way ANOVA *p* values across all groups: Gpsm2 *p*=0.088; CD5l *p*=0.025; Hhip *p*=0.0003; Tlr13 *p*=0.047; Clec3a *p*=0.166; PPAR-gamma *p*<0.0001; Lcn2 *p*<0.0001; Ucp1 *p*=0.014; Cxcl13 *p*=0.097; Ckmt2 *p*=0.018. y-axis = relative expression in arbitrary units. The Taqman analyses were performed on individual samples and each dot on the graphs represents the gene expression in the aorta from one mouse. (JPEG 44 KB)

Additional file 4: Table S2: Gene Set Enrichment Analysis. Positive enrichment: chronic *P. gingivalis-*treated group vs. chronic control group. **Table S3.** Gene Set Enrichment Analysis. Negative enrichment: chronic *P. gingivali-s*treated group vs. chronic control group. **Table S4.** Gene Set Enrichment Analysis**.** Positive enrichment: chronic *C. pneumoniae*-treated group vs. chronic control group. **Table S5.** Gene Set Enrichment Analysis. Negative enrichment: chronic *C. pneumoniae*-treated group vs. chronic control group. **Table S6.** Gene Set Enrichment Analysis. Positive enrichment: Western diet group vs. chronic control group. **Table S7.** Gene Set Enrichment Analysis. Negative enrichment: Western diet group vs. chronic control group. (PDF 244 KB)

Additional file 5: Figure S3: Chronic time point cluster analysis. **A**. DAVID analysis of chronic time point clusters 2, 4, and 5. Gene enrichment is indicated by *p* values (EASE scores, a modified Fisher exact *p* value). **B**. Box and whisker plots of the mean expression (log2) for Clusters 2, 4, and 5 reflect patterns seen on heat map. *p < 0.003 chronic treatment group vs. chronic control group; ***p < 0.0001 chronic treatment group vs. chronic control group by Mann-Whitney test. (JPEG 70 KB)

Additional file 6: **GeneLists.** Each excel sheet within this file contains gene lists corresponding to the named figure. In addition, there are two sheets that list the top 500 differentially-expressed genes: chronic time point, acute vs. chronic time point. (XLS 13 MB)

Additional file 7: Figure S4: Acute to chronic time point cluster analysis. **A**. DAVID analysis of acute to chronic time point clusters 1, 3, and 4. Gene enrichment is indicated by *p* values (EASE scores, a modified Fisher exact *p* value). **B**. Box and whisker plots of the mean expression (log2) for Clusters 1, 3, and 4 reflect patterns seen on heat map. *** = *p* < 0.0001 vs. acute control; ### = *p* < 0.0001 vs. chronic control; +++ = *p* < 0.0001 vs. acute treatment by Mann-Whitney test. (JPEG 83 KB)

Additional file 8: Figure S5-1: Acute time point cluster analysis. The top 1000 differentially expressed genes at the acute time point with 5 distinct clusters. **A**. Heat map shows relative expression among all groups. Clusters are color-coded by row sidebars: red (cluster 1), chartreuse (cluster 2), mint green (cluster 3), blue (cluster 4), and magenta (cluster 5); and dendrogram is left of the color-coded sidebars. Each row corresponds to a gene (gene symbols are listed to the right of each row) and each column to a sample. The colors are scaled by row; red and blue indicate 2 standard deviations above or below the mean (white), respectively. At the arbitrary cutoff of 1000 genes, the acute time point one-way ANOVA *p* value was < 1.5 × 10^-2^. **B**. DAVID analysis of clusters 1 and 5. Gene enrichment is indicated by *p* values (EASE scores, a modified Fisher exact *p* value). **C**. Box and whisker plots of the mean expression (log2) for Clusters 1 and 5 reflect patterns seen on heat map. ***p < 0.0001 acute treatment group vs. acute control group; +++ *p* < 0.0001 vs. *P. gingivalis* by Mann-Whitney test. (JPEG 114 KB)

Additional file 9: Figure S5-2: Acute time point cluster analysis. **A**. DAVID analysis of acute time point clusters 2, 3, and 4. Gene enrichment is indicated by *p* values (EASE scores, a modified Fisher exact *p* value) **B**. Box and whisker plots of the mean expression (log2) for Clusters 2, 3, and 4 reflect patterns seen on heat map. ***p < 0.0001 acute treatment group vs. acute control group; +++*p* < 0.0001 vs. *P. gingivalis* by Mann-Whitney test. (JPEG 59 KB)

Additional file 10: Figure S6: Genes associated with unstable plaque. Individual expression values for each sample for genes associated with unstable plaque as identified by Chen et al. [32]. Acute control group vs. chronic control group: *p < 0.05; **p < 0.01, *p* < 0.001 by Student’s t-test. Chronic control group vs. chronic treatment group: #*p* < 0.05, ##*p* < 0.01, ###*p* < 0.001 by Student’s t-test. (JPEG 2 MB)

Additional file 11: Figure S7: Experimental protocol. (JPEG 44 KB)

## References

[1] Hansson GK (2005). Inflammation, atherosclerosis, and coronary artery disease. N Engl J Med.

[2] Kronzon I, Tunick PA (2006). Aortic atherosclerotic disease and stroke. Circulation.

[3] Puri R, Nissen SE, Libby P, Shao M, Ballantyne CM, Barter PJ, Chapman MJ, Erbel R, Raichlen JS, Uno K, Kataoka Y, Nicholls SJ (2013). C-reactive protein, but not low-density lipoprotein cholesterol levels, associate with coronary atheroma regression and cardiovascular events after maximally intensive statin therapy. Circulation.

[4] Fleg JL, Forman DE, Berra K, Bittner V, Blumenthal JA, Chen MA, Cheng S, Kitzman DW, Maurer MS, Rich MW, Shen WK, Williams MA, Zieman SJ (2013). Secondary prevention of atherosclerotic cardiovascular disease in older adults: a scientific statement from the American Heart Association. Circulation.

[5] Kiechl S, Egger G, Mayr M, Wiedermann CJ, Bonora E, Oberhollenzer F, Muggeo M, Xu Q, Wick G, Poewe W, Willeit J (2001). Chronic infections and the risk of carotid atherosclerosis: prospective results from a large population study. Circulation.

[6] Li L, Messas E, Batista EL, Levine RA, Amar S (2002). Porphyromonas gingivalis infection accelerates the progression of atherosclerosis in a heterozygous apolipoprotein E-deficient murine model. Circulation.

[7] Maekawa T, Takahashi N, Tabeta K, Aoki Y, Miyashita H, Miyauchi S, Miyazawa H, Nakajima T, Yamazaki K (2011). Chronic oral infection with Porphyromonas gingivalis accelerates atheroma formation by shifting the lipid profile. PLoS One.

[8] Hayashi C, Papadopoulos G, Gudino CV, Weinberg EO, Barth KR, Madrigal AG, Chen Y, Ning H, LaValley M, Gibson FC, Hamilton JA, Genco CA (2012). Protective role for TLR4 signaling in atherosclerosis progression as revealed by infection with a common oral pathogen. J Immunol.

[9] Gibson FC, Hong C, Chou HH, Yumoto H, Chen J, Lien E, Wong J, Genco CA (2004). Innate immune recognition of invasive bacteria accelerates atherosclerosis in apolipoprotein E-deficient mice. Circulation.

[10] Hayashi C, Viereck J, Hua N, Phinikaridou A, Madrigal AG, Gibson FC, Hamilton JA, Genco CA (2011). Porphyromonas gingivalis accelerates inflammatory atherosclerosis in the innominate artery of ApoE deficient mice. Atherosclerosis.

[11] Chen S, Shimada K, Zhang W, Huang G, Crother TR, Arditi M (2010). IL-17A is proatherogenic in high-fat diet-induced and Chlamydia pneumoniae infection-accelerated atherosclerosis in mice. J Immunol.

[12] Naiki Y, Sorrentino R, Wong MH, Michelsen KS, Shimada K, Chen S, Yilmaz A, Slepenkin A, Schroder NW, Crother TR, Bulut Y, Doherty TM, Bradley M, Shaposhnik Z, Peterson EM, Tontonoz P, Shah PK, Arditi M (2008). TLR/MyD88 and liver X receptor alpha signaling pathways reciprocally control Chlamydia pneumoniae-induced acceleration of atherosclerosis. J Immunol.

[13] Campbell LA, Lee AW, Rosenfeld ME, Kuo CC (2013). Chlamydia pneumoniae induces expression of pro-atherogenic factors through activation of the lectin-like oxidized LDL receptor-1. Pathog Dis.

[14] Player MS, Mainous AG, Everett CJ, Diaz VA, Knoll ME, Wright RU (2012). Chlamydia pneumoniae and progression of subclinical atherosclerosis. Eur J Prev Cardiol.

[15] Kreutmayer S, Csordas A, Kern J, Maass V, Almanzar G, Offterdinger M, Ollinger R, Maass M, Wick G (2013). Chlamydia pneumoniae infection acts as an endothelial stressor with the potential to initiate the earliest heat shock protein 60-dependent inflammatory stage of atherosclerosis. Cell Stress Chaperones.

[16] Honarmand H (2013). Atherosclerosis Induced by Chlamydophila pneumoniae: a controversial theory. Interdiscip Perspect Infect Dis.

[17] Desvarieux M, Demmer RT, Rundek T, Boden-Albala B, Jacobs DR, Sacco RL, Papapanou PN (2005). Periodontal microbiota and carotid intima-media thickness: the Oral Infections and Vascular Disease Epidemiology Study (INVEST). Circulation.

[18] Padilla C, Lobos O, Hubert E, Gonzalez C, Matus S, Pereira M, Hasbun S, Descouvieres C (2006). Periodontal pathogens in atheromatous plaques isolated from patients with chronic periodontitis. J Periodontal Res.

[19] Haraszthy VI, Zambon JJ, Trevisan M, Zeid M, Genco RJ (2000). Identification of periodontal pathogens in atheromatous plaques. J Periodontol.

[20] Modi DK, Chopra VS, Bhau U (2009). Rheumatoid arthritis and periodontitis: biological links and the emergence of dual purpose therapies. Indian J Dent Res.

[21] Hajishengallis G (2009). Porphyromonas gingivalis-host interactions: open war or intelligent guerilla tactics?. Microbes Infect.

[22] Darveau RP, Hajishengallis G, Curtis MA (2012). Porphyromonas gingivalis as a potential community activist for disease. J Dent Res.

[23] Gibson FC, Ukai T, Genco CA (2008). Engagement of specific innate immune signaling pathways during Porphyromonas gingivalis induced chronic inflammation and atherosclerosis. Front Biosci.

[24] Hayashi C, Gudino CV, Gibson FC, Genco CA (2010). Review: Pathogen-induced inflammation at sites distant from oral infection: bacterial persistence and induction of cell-specific innate immune inflammatory pathways. Mol Oral Microbiol.

[25] **Chlamydophila pneumoniae infection. Centers for Disease Control** http://www.cdc.gov/pneumonia/atypical/chlamydophila.html

[26] Papadodima O, Sirsjo A, Kolisis FN, Chatziioannou A (2012). Application of an integrative computational framework in trancriptomic data of atherosclerotic mice suggests numerous molecular players. Adv Bioinformatics.

[27] Jawien J (2012). The role of an experimental model of atherosclerosis: apoE-knockout mice in developing new drugs against atherogenesis. Curr Pharm Biotechnol.

[28] Jawien J, Nastalek P, Korbut R (2004). Mouse models of experimental atherosclerosis. J Physiol Pharmacol.

[29] Subramanian A, Tamayo P, Mootha VK, Mukherjee S, Ebert BL, Gillette MA, Paulovich A, Pomeroy SL, Golub TR, Lander ES, Mesirov JP (2005). Gene set enrichment analysis: a knowledge-based approach for interpreting genome-wide expression profiles. Proc Natl Acad Sci U S A.

[30] Mootha VK, Lindgren CM, Eriksson KF, Subramanian A, Sihag S, Lehar J, Puigserver P, Carlsson E, Ridderstrale M, Laurila E, Houstis N, Daly MJ, Patterson N, Mesirov JP, Golub TR, Tamayo P, Spiegelman B, Lander ES, Hirschhorn JN, Altshuler D, Groop LC (2003). PGC-1alpha-responsive genes involved in oxidative phosphorylation are coordinately downregulated in human diabetes. Nat Genet.

[31] Subramanian A, Kuehn H, Gould J, Tamayo P, Mesirov JP (2007). GSEA-P: a desktop application for Gene Set Enrichment Analysis. Bioinformatics.

[32] Chen YC, Bui AV, Diesch J, Manasseh R, Hausding C, Rivera J, Haviv I, Agrotis A, Htun NM, Jowett J, Hagemeyer CE, Hannan RD, Bobik A, Peter K (2013). A novel mouse model of atherosclerotic plaque instability for drug testing and mechanistic/therapeutic discoveries using gene and microRNA expression profiling. Circ Res.

[33] Moore KJ, Sheedy FJ, Fisher EA (2013). Macrophages in atherosclerosis: a dynamic balance. Nat Rev Immunol.

[34] Edfeldt K, Swedenborg J, Hansson GK, Yan ZQ (2002). Expression of toll-like receptors in human atherosclerotic lesions: a possible pathway for plaque activation. Circulation.

[35] Xu XH, Shah PK, Faure E, Equils O, Thomas L, Fishbein MC, Luthringer D, Xu XP, Rajavashisth TB, Yano J, Kaul S, Arditi M (2001). Toll-like receptor-4 is expressed by macrophages in murine and human lipid-rich atherosclerotic plaques and upregulated by oxidized LDL. Circulation.

[36] Bjorkbacka H, Kunjathoor VV, Moore KJ, Koehn S, Ordija CM, Lee MA, Means T, Halmen K, Luster AD, Golenbock DT, Freeman MW (2004). Reduced atherosclerosis in MyD88-null mice links elevated serum cholesterol levels to activation of innate immunity signaling pathways. Nat Med.

[37] Higashimori M, Tatro JB, Moore KJ, Mendelsohn ME, Galper JB, Beasley D (2011). Role of toll-like receptor 4 in intimal foam cell accumulation in apolipoprotein E-deficient mice. Arterioscler Thromb Vasc Biol.

[38] Michelsen KS, Wong MH, Shah PK, Zhang W, Yano J, Doherty TM, Akira S, Rajavashisth TB, Arditi M (2004). Lack of Toll-like receptor 4 or myeloid differentiation factor 88 reduces atherosclerosis and alters plaque phenotype in mice deficient in apolipoprotein E. Proc Natl Acad Sci U S A.

[39] Mullick AE, Tobias PS, Curtiss LK (2005). Modulation of atherosclerosis in mice by Toll-like receptor 2. J Clin Invest.

[40] Liu X, Ukai T, Yumoto H, Davey M, Goswami S, Gibson FC, Genco CA (2008). Toll-like receptor 2 plays a critical role in the progression of atherosclerosis that is independent of dietary lipids. Atherosclerosis.

[41] Jain S, Coats SR, Chang AM, Darveau RP (2013). A novel class of lipoprotein lipase-sensitive molecules mediates Toll-like receptor 2 activation by Porphyromonas gingivalis. Infect Immun.

[42] Nichols FC, Bajrami B, Clark RB, Housley W, Yao X (2012). Free lipid A isolated from Porphyromonas gingivalis lipopolysaccharide is contaminated with phosphorylated dihydroceramide lipids: recovery in diseased dental samples. Infect Immun.

[43] Miller SI, Ernst RK, Bader MW (2005). LPS, TLR4 and infectious disease diversity. Nat Rev Microbiol.

[44] Coats SR, Pham TT, Bainbridge BW, Reife RA, Darveau RP (2005). MD-2 mediates the ability of tetra-acylated and penta-acylated lipopolysaccharides to antagonize Escherichia coli lipopolysaccharide at the TLR4 signaling complex. J Immunol.

[45] Reife RA, Coats SR, Al-Qutub M, Dixon DM, Braham PA, Billharz RJ, Howald WN, Darveau RP (2006). Porphyromonas gingivalis lipopolysaccharide lipid A heterogeneity: differential activities of tetra- and penta-acylated lipid A structures on E-selectin expression and TLR4 recognition. Cell Microbiol.

[46] Slocum C, Coats SR, Hua N, Kramer C, Papadopoulos G, Weinberg EO, Gudino CV, Hamilton JA, Darveau RP, Genco CA (2014). Distinct lipid a moieties contribute to pathogen-induced site-specific vascular inflammation. PLoS Pathog.

[47] Prebeck S, Kirschning C, Durr S, da Costa C, Donath B, Brand K, Redecke V, Wagner H, Miethke T (2001). Predominant role of toll-like receptor 2 versus 4 in Chlamydia pneumoniae-induced activation of dendritic cells. J Immunol.

[48] Beatty WL, Morrison RP, Byrne GI (1994). Persistent chlamydiae: from cell culture to a paradigm for chlamydial pathogenesis. Microbiol Rev.

[49] Blasi F, Centanni S, Allegra L (2004). Chlamydia pneumoniae: crossing the barriers?. Eur Respir J.

[50] Kalayoglu MV, Indrawati ᅟ, Morrison RP, Morrison SG, Yuan Y, Byrne GI (2000). Chlamydial virulence determinants in atherogenesis: the role of chlamydial lipopolysaccharide and heat shock protein 60 in macrophage-lipoprotein interactions. J Infect Dis.

[51] Cao F, Castrillo A, Tontonoz P, Re F, Byrne GI (2007). Chlamydia pneumoniae–induced macrophage foam cell formation is mediated by Toll-like receptor 2. Infect Immun.

[52] Bjorkegren JL, Hagg S, Talukdar HA, Foroughi Asl H, Jain RK, Cedergren C, Shang MM, Rossignoli A, Takolander R, Melander O, Hamsten A, Michoel T, Skogsberg J (2014). Plasma cholesterol-induced lesion networks activated before regression of early, mature, and advanced atherosclerosis. PLoS Genet.

[53] Madamanchi NR, Runge MS (2007). Mitochondrial dysfunction in atherosclerosis. Circ Res.

[54] Phinikaridou A, Andia ME, Passacquale G, Ferro A, Botnar RM (2013). Noninvasive MRI monitoring of the effect of interventions on endothelial permeability in murine atherosclerosis using an albumin-binding contrast agent. J Am Heart Assoc.

[55] Erridge C (2010). Endogenous ligands of TLR2 and TLR4: agonists or assistants?. J Leukoc Biol.

[56] Weinberg EO, Genco CA (2012). Directing TRAF-ic: cell-specific TRAF6 signaling in chronic inflammation and atherosclerosis. Circulation.

[57] Hyvarinen K, Tuomainen AM, Laitinen S, Alfthan G, Salminen I, Leinonen M, Saikku P, Kovanen PT, Jauhiainen M, Pussinen PJ (2013). The effect of proatherogenic pathogens on adipose tissue transcriptome and fatty acid distribution in apolipoprotein E-deficient mice. BMC Genomics.

[58] Hayashi C, Madrigal AG, Liu X, Ukai T, Goswami S, Gudino CV, Gibson FC, Genco CA (2010). Pathogen-mediated inflammatory atherosclerosis is mediated in part via Toll-like receptor 2-induced inflammatory responses. J Innate Immun.

[59] Cao C, Zhu Y, Chen W, Li L, Qi Y, Wang X, Zhao Y, Wan X, Chen X (2013). IKKepsilon knockout prevents high fat diet induced arterial atherosclerosis and NF-kappaB signaling in mice. PLoS One.

[60] Lee J, Baldwin WM, Lee CY, Desiderio S (2013). Stat3beta mitigates development of atherosclerosis in apolipoprotein E-deficient mice. J Mol Med (Berl).

[61] Wang H, Zhu HQ, Wang F, Zhou Q, Gui SY, Wang Y (2013). MicroRNA-1 prevents high-fat diet-induced endothelial permeability in apoE knock-out mice. Mol Cell Biochem.

[62] Pi X, Lockyer P, Dyer LA, Schisler JC, Russell B, Carey S, Sweet DT, Chen Z, Tzima E, Willis MS, Homeister JW, Moser M, Patterson C (2012). Bmper inhibits endothelial expression of inflammatory adhesion molecules and protects against atherosclerosis. Arterioscler Thromb Vasc Biol.

[63] Moazed TC, Campbell LA, Rosenfeld ME, Grayston JT, Kuo CC (1999). Chlamydia pneumoniae infection accelerates the progression of atherosclerosis in apolipoprotein E-deficient mice. J Infect Dis.

[64] Moazed TC, Kuo C, Grayston JT, Campbell LA (1997). Murine models of Chlamydia pneumoniae infection and atherosclerosis. J Infect Dis.

[65] Papadopoulos G, Kramer CD, Slocum CS, Weinberg EO, Hua N, Gudino CV, Hamilton JA, Genco CA (2014). A mouse model for pathogen-induced chronic inflammation at local and systemic sites. J Vis Exp.

[66] He X, Mekasha S, Mavrogiorgos N, Fitzgerald KA, Lien E, Ingalls RR (2010). Inflammation and fibrosis during Chlamydia pneumoniae infection is regulated by IL-1 and the NLRP3/ASC inflammasome. J Immunol.

[67] He X, Nair A, Mekasha S, Alroy J, O'Connell CM, Ingalls RR (2011). Enhanced virulence of Chlamydia muridarum respiratory infections in the absence of TLR2 activation. PLoS One.

[68] Storey JD, Tibshirani R (2003). Statistical significance for genomewide studies. Proc Natl Acad Sci U S A.

[69] Storey JD, Tibshirani R (2003). Statistical methods for identifying differentially expressed genes in DNA microarrays. Methods Mol Biol.

[70] Smyth GK (2004). Linear models and empirical bayes methods for assessing differential expression in microarray experiments. Stat Appl Genet Mol Biol.

[71] Huang da W, Sherman BT, Zheng X, Yang J, Imamichi T, Stephens R, Lempicki RA (2012). Extracting biological meaning from large gene lists with DAVID. Curr Protoc Bioinformatics.

[72] da Huang W, Sherman BT, Lempicki RA (2009). Systematic and integrative analysis of large gene lists using DAVID bioinformatics resources. Nat Protoc.

[73] Kramer CD, Weinberg EO (2014). Genome Data from Series Record ID GSE60086.

