# Supplementary figures and images for: Distinct gene signatures in aortic tissue from ApoE-/- mice exposed to pathogens or Western diet
Source: BMC Genomics. 2014 Dec 24;15(1):1176. doi: 10.1186/1471-2164-15-1176 (PMC4367889; doi:10.1186/1471-2164-15-1176)

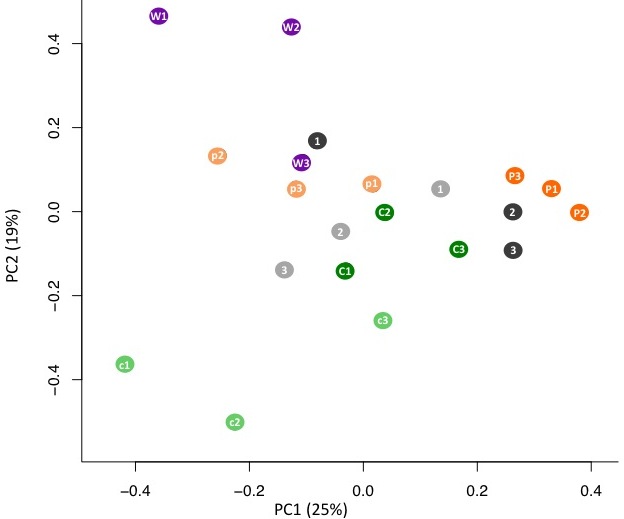

Supplement: Supplementary file 1 — Additional file 1: Figure S1: Principal Component Analysis (PCA). Graph showing variance in global expression in each individual sample array in relation to all 21 arrays. The first principal component (PC1, x axis) had a variance of 25% and the second principal component (PC2, y axis) had a variance of 19%. Note that the 3 replicates in each group cluster near each other. Acute control group: light grey 1, 2, 3; chronic control group: dark grey 1, 2, 3; acute P. gingivalis-treated group: light orange p1, p2, p3; chronic P. gingivalis-treated group: dark orange P1, P2, P3; acute C. pneumoniae-treated group: light green c1, c2, c3; chronic C. pneumoniae-treated group: dark green C1, C2, C3; WD group: purple W1, W2, W3. The group with the largest variance among the three replicates is the acute C. pneumoniae-treated group. (JPEG 27 KB) [file 12864_2014_6916_MOESM1_ESM.jpeg]

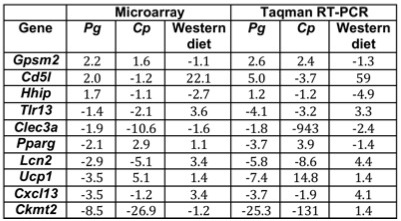

Supplement: Supplementary file 2 — Additional file 2: Table S1: Comparison of microarray and RT-PCR expression results for 10 genes. Comparison of mean fold changes in gene expression obtained by microarray analysis and real time RT-PCR for 10 genes at the chronic time point. P. gingivalis = P. gingivalis-treated group; C. pneumoniae = C. pneumoniae-treated group. (JPEG 45 KB) [file 12864_2014_6916_MOESM2_ESM.jpeg]

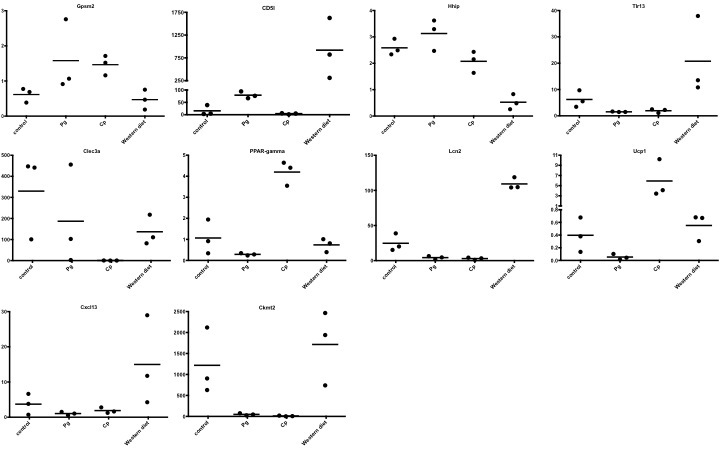

Supplement: Supplementary file 3 — Additional file 3: Figure S2: Taqman validation of 10 genes. One-way ANOVA p values across all groups: Gpsm2 p=0.088; CD5l p=0.025; Hhip p=0.0003; Tlr13 p=0.047; Clec3a p=0.166; PPAR-gamma p<0.0001; Lcn2 p<0.0001; Ucp1 p=0.014; Cxcl13 p=0.097; Ckmt2 p=0.018. y-axis = relative expression in arbitrary units. The Taqman analyses were performed on individual samples and each dot on the graphs represents the gene expression in the aorta from one mouse. (JPEG 44 KB) [file 12864_2014_6916_MOESM3_ESM.jpeg]

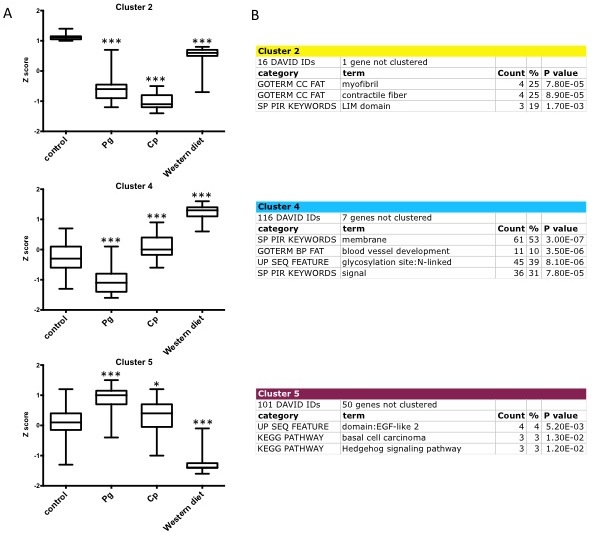

Supplement: Supplementary file 5 — Additional file 5: Figure S3: Chronic time point cluster analysis. A. DAVID analysis of chronic time point clusters 2, 4, and 5. Gene enrichment is indicated by p values (EASE scores, a modified Fisher exact p value). B. Box and whisker plots of the mean expression (log2) for Clusters 2, 4, and 5 reflect patterns seen on heat map. *p < 0.003 chronic treatment group vs. chronic control group; ***p < 0.0001 chronic treatment group vs. chronic control group by Mann-Whitney test. (JPEG 70 KB) [file 12864_2014_6916_MOESM5_ESM.jpeg]

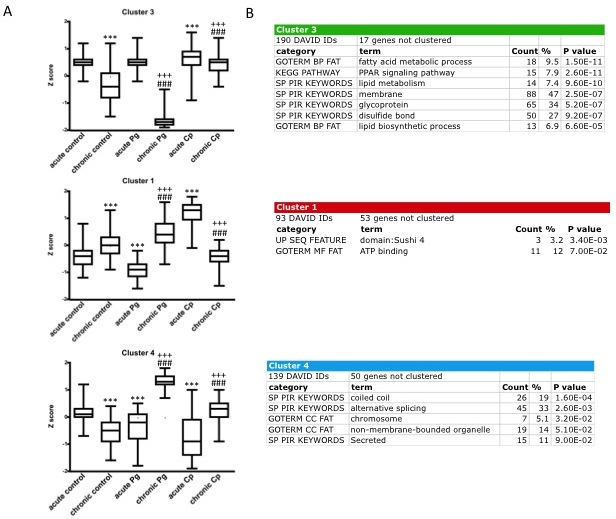

Supplement: Supplementary file 7 — Additional file 7: Figure S4: Acute to chronic time point cluster analysis. A. DAVID analysis of acute to chronic time point clusters 1, 3, and 4. Gene enrichment is indicated by p values (EASE scores, a modified Fisher exact p value). B. Box and whisker plots of the mean expression (log2) for Clusters 1, 3, and 4 reflect patterns seen on heat map. *** = p < 0.0001 vs. acute control; ### = p < 0.0001 vs. chronic control; +++ = p < 0.0001 vs. acute treatment by Mann-Whitney test. (JPEG 83 KB) [file 12864_2014_6916_MOESM7_ESM.jpeg]

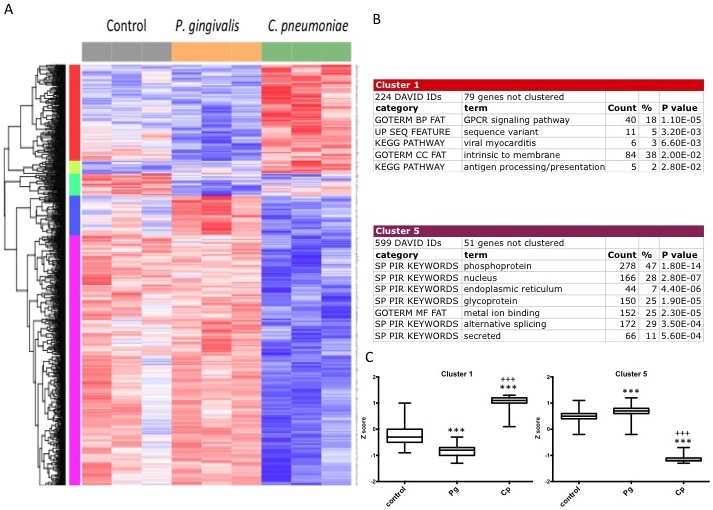

Supplement: Supplementary file 8 — Additional file 8: Figure S5-1: Acute time point cluster analysis. The top 1000 differentially expressed genes at the acute time point with 5 distinct clusters. A. Heat map shows relative expression among all groups. Clusters are color-coded by row sidebars: red (cluster 1), chartreuse (cluster 2), mint green (cluster 3), blue (cluster 4), and magenta (cluster 5); and dendrogram is left of the color-coded sidebars. Each row corresponds to a gene (gene symbols are listed to the right of each row) and each column to a sample. The colors are scaled by row; red and blue indicate 2 standard deviations above or below the mean (white), respectively. At the arbitrary cutoff of 1000 genes, the acute time point one-way ANOVA p value was < 1.5 × 10^-2^. B. DAVID analysis of clusters 1 and 5. Gene enrichment is indicated by p values (EASE scores, a modified Fisher exact p value). C. Box and whisker plots of the mean expression (log2) for Clusters 1 and 5 reflect patterns seen on heat map. ***p < 0.0001 acute treatment group vs. acute control group; +++ p < 0.0001 vs. P. gingivalis by Mann-Whitney test. (JPEG 114 KB) [file 12864_2014_6916_MOESM8_ESM.jpeg]

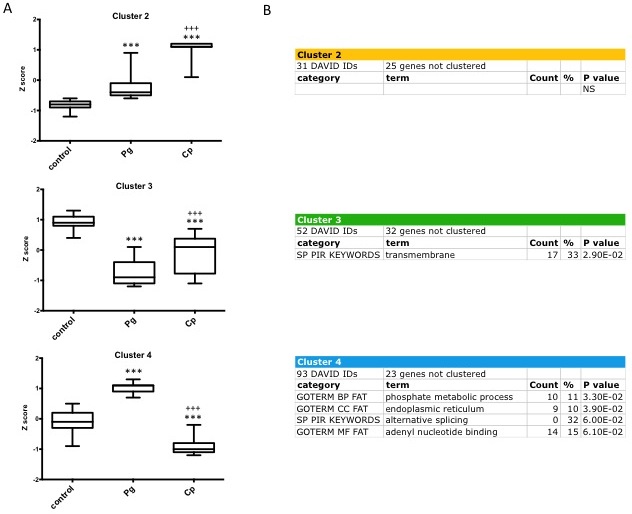

Supplement: Supplementary file 9 — Additional file 9: Figure S5-2: Acute time point cluster analysis. A. DAVID analysis of acute time point clusters 2, 3, and 4. Gene enrichment is indicated by p values (EASE scores, a modified Fisher exact p value) B. Box and whisker plots of the mean expression (log2) for Clusters 2, 3, and 4 reflect patterns seen on heat map. ***p < 0.0001 acute treatment group vs. acute control group; +++p < 0.0001 vs. P. gingivalis by Mann-Whitney test. (JPEG 59 KB) [file 12864_2014_6916_MOESM9_ESM.jpeg]

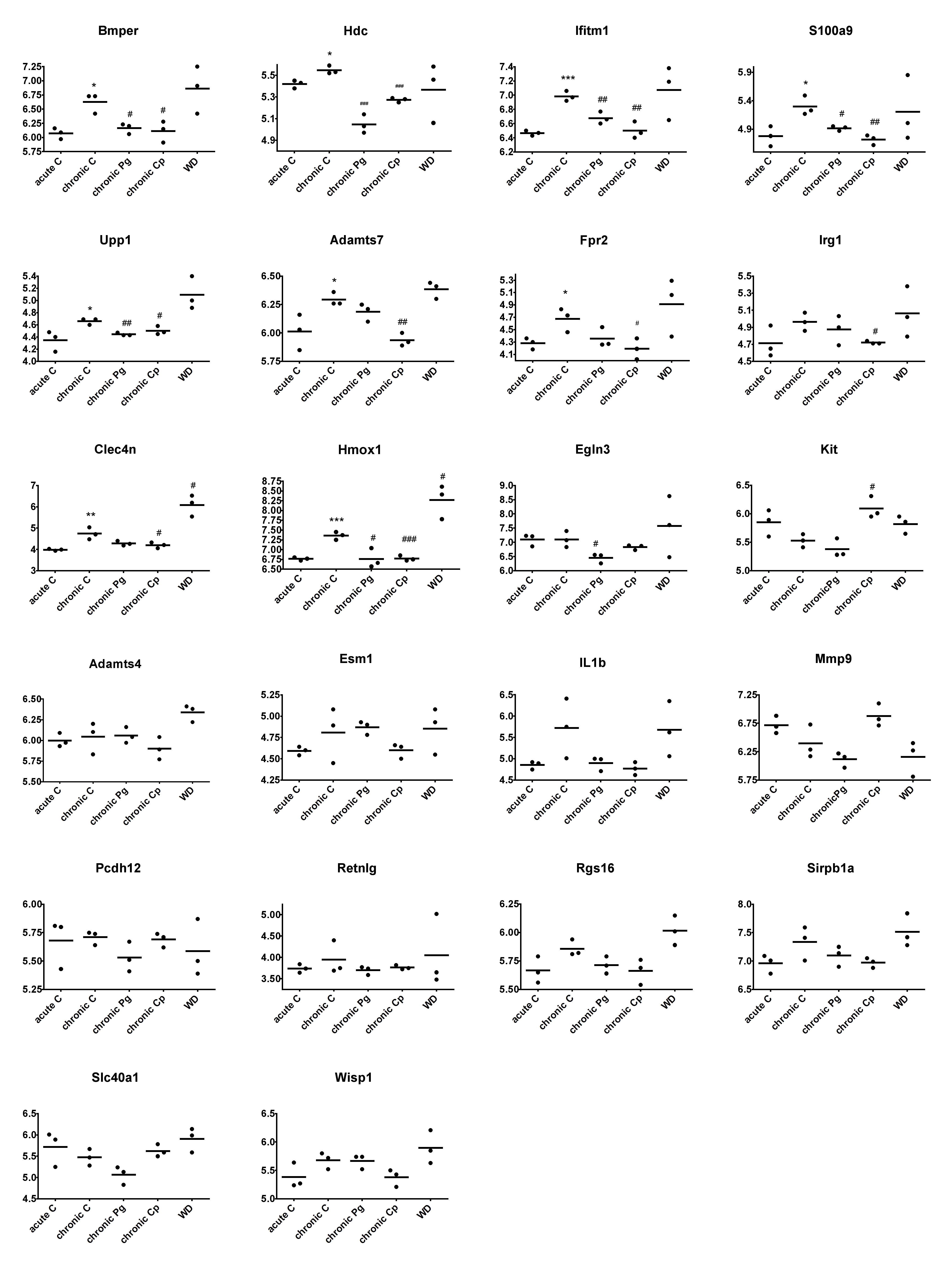

Supplement: Supplementary file 10 — Additional file 10: Figure S6: Genes associated with unstable plaque. Individual expression values for each sample for genes associated with unstable plaque as identified by Chen et al. [32]. Acute control group vs. chronic control group: *p < 0.05; **p < 0.01, p < 0.001 by Student’s t-test. Chronic control group vs. chronic treatment group: #p < 0.05, ##p < 0.01, ###p < 0.001 by Student’s t-test. (JPEG 2 MB) [file 12864_2014_6916_MOESM10_ESM.jpeg]

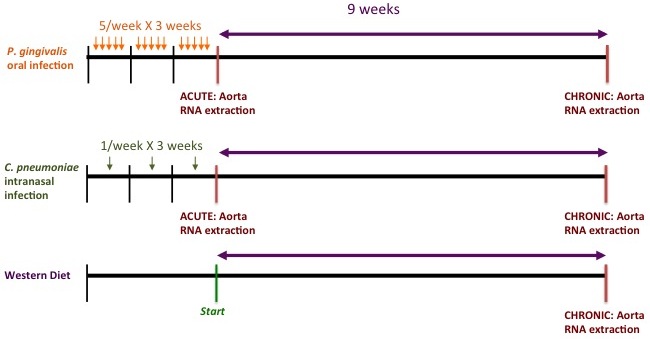

Supplement: Supplementary file 11 — Additional file 11: Figure S7: Experimental protocol. (JPEG 44 KB) [file 12864_2014_6916_MOESM11_ESM.jpeg]
