# Supplementary material for: Distinct gene signatures in aortic tissue from ApoE-/- mice exposed to pathogens or Western diet
Source: BMC Genomics. 2014 Dec 24;15(1):1176. doi: 10.1186/1471-2164-15-1176 (PMC4367889; doi:10.1186/1471-2164-15-1176)
Supplement: Supplementary file 4 — Additional file 4: Table S2: Gene Set Enrichment Analysis. Positive enrichment: chronic P. gingivalis-treated group vs. chronic control group. Table S3. Gene Set Enrichment Analysis. Negative enrichment: chronic P. gingivali-streated group vs. chronic control group. Table S4. Gene Set Enrichment Analysis. Positive enrichment: chronic C. pneumoniae-treated group vs. chronic control group. Table S5. Gene Set Enrichment Analysis. Negative enrichment: chronic C. pneumoniae-treated group vs. chronic control group. Table S6. Gene Set Enrichment Analysis. Positive enrichment: Western diet group vs. chronic control group. Table S7. Gene Set Enrichment Analysis. Negative enrichment: Western diet group vs. chronic control group. (PDF 244 KB) [file 12864_2014_6916_MOESM4_ESM.pdf]

Supplemental Table 2 - Gene Set Enrichment Analysis. Positive enrichment: chronic *P. gingivalis*-treated group vs. chronic control group

Gene Set	Genes Contributing to Core Enrichment
**1 BASAL CELL CARCINOMA**	Trp53, Dvl2, Gli3, Wnt5b, Smo, Ctnnb1, Fzd1, Fzd6, Tcf7, Sufu, Fzd8, Ptch2, Gli1, Fzd5, Ptch1, Wnt16, Gli2,<br>Wnt5a, Hhip, Tcf7l1, Wnt9a, Tcf7l2, Fzd2, Bmp4, Fzd7
**3 NUCLEACHROMOSOME PART**	H2afy, Cbx1, H1fnt, Pura, Syce1, Syce2, Ercc4, Atrx, Ube2i, Tipin, Pola1, Cbx5, 1110036O03Rik, Foxc1, H2afy2
**5 REGULATION OF GENE EXPRESSION EPIGENETIC**	Gpx1, Crebzf, Dmap1, Tnp1, H2afy, Dnmt1, Dicer1, Hmga1, Dgcr8, Rag1ap1, Smarcd1, Atf7ip, Atrx, Dnmt3a,<br>Glmn, H2afy2
**6 KEGG MELANOGENESIS**	Mc1r, Dvl2, Camk2d, A, Wnt5b, Creb3l4, Ctnnb1, Fzd1, Gnai2, Adcy5, Fzd6, Tcf7, Gnaq, Creb3l2, Creb3l1, Plcb3, Fzd8, Adcy7, Mapk3, Fzd5, Wnt16, Adcy3, Prkacb, Plcb1, Gnai3, Wnt5a, Gnai1, Tcf7l1, Camk2b, Plcb4,<br>Wnt9a, Camk2g, Tcf7l2, Fzd2, Fzd7
**8 DNA REPLICATION**<br>2 DNA DEPENDENT DNA REPLICATION<br>11 REGULATION OF DNA REPLICATION	[2]Orc2l, Rev1, [2]Orc5l, [2]Pura, [2,11]Enpp7, [2]Rev3l, Pole3, Pot1a, [2]Msh2, Terf2, [2,11]Cdt1, [2]Msh6, Nae1, [2]Mlh1, [2,11]Nbn, [2]Polg2, Pold4, Terf2ip, [2]Rfc1, Rad50, [2,11]Ccdc88a, Nap1l1, Pole2, [2]Prim2, [2,11]S100a11, [2,11]Gli1, [2]Pms2, [2,11]Gli2, Ptms, [2,11]Tipin, [2]Pola1, [2]Abl1, [2]Mutyh, [2]Orc4l, [2]Prim1
**9 PROTEINACEOUS EXTRACELLULAR MATRIX**<br>4 EXTRACELLULAR MATRIX PART<br>7 BASEMENT MEMBRANE<br>13 EXTRACELLULAR MATRIX	[4,7]Fbn1, [4]Col5a1, [4,7]Col4a3, Adamts5, [4,7]Lama4, [4,7]Col4a4, Prelp, [4]Col18a1, [4,7]Smc3, [4,7]Lamb2, [4]Col5a2, [4]Tnxb, [4,7]Magee1, Ltbp4, [4,7]Aplp1, [4,7]Agrn, [4]Col6a3, [4]Col3a1, [4]Col1a2, [4]Col15a1, [4,7]Efemp2, [4,7]Sntb2, Fbln5, Optc, Postn, [4,7]Sgce, Mmp11, Ecm2, [4,7]Dmd, [4,7]Sgcd, [4,7]Sntg2, [4,7]Sntb1, [4,7]Col4a5
**10 KEGG HEDGEHOG SIGNALING PATHWAY**	Gas1, Csnk1g3, Ihh, Gsk3b, Rab23, Gli3, Wnt5b, Smo, Csnk1a1, Csnk1g1, Sufu, Fbxw11, Ptch2, Gli1, Ptch1,<br>Wnt16, Gli2, Prkacb, Wnt5a, Hhip, Wnt9a, Bmp4
**12 REACTOME CELL EXTRACELLULAR MATRIX INTERACTIONS**	Vasp, Fermt2, Itgb1, Parva, Lims2, Flna, Actn1, Rsu1, Fblim1, Ilk
**14 REACTOME E2F MEDIATED REGULATION OF DNA REPLICATION**	Dhfr, Orc2l, Orc5l, Ccna1, Cdc25a, Cdt1, Fbxo5, Pou5f1, Rrm2, Prim2, Tk2, Pola1, Orc4l, Prim1
**15 REACTOME APOPTOTIC EXECUTION PHASE**	Bcap31, Hist1h1c, Add1, 2610018G03Rik, H1f0, Vim, Dbnl, Ctnnb1, Casp3, Prkcd, Casp8, Dsg3, Pak2, Hist1h1b, Stk24, Hist1h1a, Dsg1b, Lmna, Tjp2, Rock1, Hist1h1e
**16 COLLAGEN**	Col11a1, Col13a1, Col16a1, Col10a1, Lum, Col7a1, Col9a2, Col9a1, Col5a1, Col4a3, Col4a4, Col18a1, Col5a2, Tnxb, Col6a3, Col3a1, Col1a2, Col15a1, Col4a5
**17 REACTOME INACTIVATION OF APC VIA DIRECT INHIBITION OF THE AP COMPLEX**	Anapc2, Cdc26, Bub3, Cdc27, Anapc5, Cdc16, Cdc23, Mad2l1, Anapc7, Anapc4
**19 REACTOME SMOOTH MUSCLE**	Acta2, Myl12b, Myl6b, Itgb5, Myl6, Mylk, Tln1, Itga1, Myh11, Lmod1, Tpm2, Vcl

**CONTRACTION**	
**20 REACTOME CONVERSION FROM APC CDC20 TO APC CD1 IN LATE ANAPHASE**	Cdc14a, Anapc4, Anapc7, Cdc23, Cdc16, Anapc5, Cdc27
**21 REACTOME SIGNALING BY PDGF**	Pik3cb, Plg, Col5a1, Col4a3, Col4a4, Col5a2, Col6a2, Pik3r2, Ywhab, Col6a1, Mapk3, Col6a3, Col3a1, Col1a2, Src, Col6a6, Plat, Pdgfc, Pdgfd, Thbs2, Pdgfa, Col4a5, Pdgfrb
**22 KEGG LYSOSOME**	Glb1, Lipa, Ap1s1, Abca2, Atp6v0d1, Ctsb, Idua, Ap1m2, Manba, Ap1s2, Aga, Gga3, Cd164, Ctso, Naglu, Tpp1, Ctsf, Sgsh, Fuca1, Gns, Arsg, Gm2a, Gga2, Atp6v1h, Gla, Cltc, Ap3m2, Abcb9, Ctsh, Sumf1, Cd63, Arsb, Slc17a5, Atp6v0d2, Ctsl, Ids, Ctsk, Asah1, Pla2g15, Ppt1, Hyal1, Hexb
**23 MICROTUBULE CYTOSKELETON**	Ranbp9, Pcm1, 6330503K22Rik, Akap9, Ccna1, 1700057K13Rik, Pkd2, Clasp2, Kif3c, Abca2, Sac3d1, Cetn3, Ckap5, Npm1, Espl1, Rpgrip1l, Tubgcp3, Brca2, Cep57, Katna1, Tsga14, Crocc, Fbxo5, Stau1, Dynll1, Cdk5rap2, Smc3, Cep63, Lyst, Cdc27, Rabgap1, Cdc16, Dnali1, Kifap3, Lats1, Mid1, Mark1, Atg4c, Gas8, Tubgcp5, Bbs4, Mtap1a, Alms1, Eml2, Ppp4r2, Sass6, Eml1, Mtap1b, Arhgef2, Sept9, Mapre1
**24 CELL DIVISION**<br>18 CYTOKINESIS	Pard3, Racgap1, Brca2, Tgfb2, [18]Anln, [18]Sept7, [18]Sept5, [18]Diap2, [18]Myh10, [18]Sept6
**25 REACTOME CLASS B2 SECRETIN FAMILY RECEPTORS**	Smo, Gngt1, Calcrl, Fzd1, Fzd6, Fzd8, Ptch2, Fzd5, Ptch1, Gng8, Wnt16, Vipr2, Wnt5a, Ramp1, Wnt9a, Gng12, Gnb4, Fzd2, Fzd7

The top 25 *P. gingivalis* gene sets whose member genes are predominantly upregulated with respect to the chronic control group. Column 1 includes the gene set name (bold) as provided by MSigDB and the rank of the gene set based on Normalized Enrichment Score (NES). Gene set names that are not in bold are gene sets that are redundant or partially redundant and included within larger bolded gene sets. The NES ranged from 2.32 (for Basal Cell Carcinoma, the top ranked gene set) to 1.66 (REACTOME Class B2 Secretin Family Receptors, the 25th-ranked gene set). FDR q range: 0.001-0.186. Column 2 lists those genes within the gene set that significantly contributed to the enrichment of the gene set. Genes within redundant gene sets are preceded by corresponding superscripts for the gene set to which they belong.

Supplemental Table 3 - Gene Set Enrichment Analysis. Negative enrichment: chronic *P. gingivalis*-treated group vs. chronic control group

Gene Set	Genes Contributing to Core Enrichment
**2 REACTOME INTEGRATION OF ENERGY METABOLISM**	Gm12251, Adcy6, Gnao1, Pdp2, Gm5506, Ndufv3, Snap25, Gpi1, Pfkfb3, Ndufb3, Pfkfb1, Ndufa6, Gng2, Ndufb6, Ndufa13, Ndufa2, Acaca, Tpi1, Pkm2, Gck, Gng11, Prkaa2, Acly, Ndufa1, Stradb, Etfa, Ndufb2, Rapgef3, Ndufa12, Pfkl, Nnt, Suclg1, Sdhc, Cox8a, Pdhx, Pfkfb2, Ndufb11, Ndufc2, Abcc8, Dld, Cox6c, Ndufs8, Ndufv2, Adcy4, Ndufa7, Ndufb7, Atp5k, Prkar2b, Acsl1, Ndufa9, Fasn, Ndufs4, Ndufa4, Atp5o, Dlst, Cox7b, Atp5j2, Atp5d, Atp5f1, Atp5j, Aldoa, Gm4943, Kcnj11, Mlxipl, Ndufb9, Sucla2, Pdk2, Cs, Uqcrc2, Idh3g, Cox4i1, Slc25a4, Idh3b, Ndufs7, Etfdh, Sdhd, Uqcr10, Sdha, Ndufs6, Ndufa5, Pdk1, Slc25a20, Rapgef4, Pcx, Ndufs1, Pdha1, Dlat, Cpt2, Idh2, Ndufc1, Atp5c1, Pdhb, Ndufb8, Idh3a, Atp5a1, Atp5b, Fh1, Pfkm, Ndufs2, Ndufa3, Ndufa8, Etfb, Uqcrfs1, Cox6b1, Ogdh, Sdhb, Ndufb5, Ndufb10, Ndufa10, Cacna2d2, Pgam2, Ndufv1, Cyc1, Uqcrc1, Acacb, Eno3, Cpt1b, Aco2, Mdh2, Pdk4, Cox5a, Uqcrq
**4 KEGG OXIDATIVE PHOSPHORYLATION**	Tcirg1, Atp6v0b, Cox4i2, Atp6v1e1, Cox6a1, Ndufs5, Ppa2, Ndufa4l2, Atp6v0a2, Atp6v1b2, Ndufv3, Cox17, Ndufb3, Ndufa6, Ndufb6, Ndufs3, Ndufa2, Ndufa1, Ndufb2, Sdhc, Cox8a, Cox7a2, Ndufa11, Ndufc2, Cox6c, Cox10, Ndufs8, Ndufv2, Ndufa7, Ndufb7, Atp5k, Ndufa9, Ndufs4, Ndufa4, Atp5o, Uqcrh, Atp5j2, Cox7b, Atp5d, Atp5f1, Atp5j, Ppa1, Ndufb9, Uqcrc2, Cox4i1, Ndufs7, Sdhd, Uqcr10, Sdha, Ndufs6, Ndufa5, Atp5g3, Ndufs1, Ndufc1, Atp5c1, Ndufb8, Atp5a1, Atp5b, Ndufs2, Ndufa3, Ndufa8, Uqcrfs1, Cox6b1, Sdhb, Ndufb5, Ndufb10, Ndufa10, Ndufv1, Cyc1, Uqcrc1, Cox7a1, Cox6a2, Cox5a, Uqcrq
**5 KEGG PARKINSONS DISEASE**	Uba7, Ndufb3, Ndufa6, Ndufb6, Vdac2, Pink1, Ndufs3, Ndufa2, Ndufa1, Ndufb2, Sdhc, Cox8a, Cox7a2, Ndufc2, Cox6c, Ndufs8, Ube2g2, Ndufv2, Ndufa7, Ndufb7, Ndufa9, Ndufs4, Ndufa4, Atp5o, Uqcrh, Cox7b, Atp5d, Atp5f1, Atp5j, Vdac1, Ndufb9, Uqcrc2, Cox4i1, Slc25a4, Ndufs7, Sdhd, Uqcr10, Sdha, Ndufs6, Ndufa5, Atp5g3, Ndufs1, Ndufc1, Atp5c1, Ndufb8, Atp5a1, Atp5b, Ndufs2, Ndufa3, Ndufa8, Uqcrfs1, Cox6b1, Sdhb, Ndufb5, Ndufb10, Ndufa10, Ndufv1, Cyc1, Uqcrc1, Cox7a1, Cox6a2, Cox5a, Uqcrq
**7 MITOCHONDRIAL PART**	Cox15, Acn9, Mrps10, Bckdk, Mrps18c, Gatm, Mrps24, Mrpl10, Mrps15, Slc25a22, Bcs1l, Timm44, Fis1, Timm50, Pin4, Mrps28, Aifm2, Ndufa6, Mrps35, Mrps18a, Mrps12, Ndufb6, Hccs, Alas2, Vdac2, Ndufa13, Opa1, Mrpl55, Ndufs3, Ndufa2, Timm8b, Aldh4a1, Ndufa1, Supv3l1, Mrpl51, Etfa, Abcb6, Ucp3, Nfs1, Abcb8, Oxa1l, Dnaja3, Phb, Nnt, Phb2, Slc25a3, Timm10, Immt, Slc25a11, Ndufs8, Grpel1, Bckdha, Ndufa9, Ndufs4, Atp5o, Atp5d, Atp5f1, Pmpca, Atp5j, Vdac1, Cs, Mrpl12, Acadm, Rhot2, Mrpl42, Ndufs7, Sdhd, Sdha, Atp5g3, Ndufs1, Mtx2, Atp5c1, Atp5a1, Mfn2, Atp5b, Ndufs2, Etfb, Ogdh, Ndufv1, Uqcrc1
**8 MITOCHONDRIAN**<br>10 MITOCHONDRIAL MEMBRANE<br>11 MITOCHONDRIAL ENVELOPE<br>17 MITOCHONDRIAL MEMBRANE PART	Hsd17b10, Bckdk, Mrps18c, Arg2, Gatm, Gstz1, Mrps24, Mrpl10, Sco1, Ndufs5, Mrps15, Gcdh, Slc25a22, Mrps31, Spg7, Slc22a4, Oxsm, [10,17]Bcs1l, Polg, Timm44, [10,17]Fis1, Cln3, Mthfd2, Letm1, Lonp1, Fdx1, Pts, Mtch1, [10,17]Timm50, Pin4, Mrps28, Pccb, 0610009O20Rik, Tsfm, Mipep, Coq6, Aifm1, [10,11]Aifm2, [10,11,17]Ndufa6, Mrps35, Mrps18a, Pgs1, Bcl2l1, Mrps12, Msrb2, [10,11,17]Ndufb6, [10,11]Hccs, Ldhd, [10,11]Alas2, Clpx, [10,11]Vdac2, [10,11,17]Ndufa13, [10,11,17]Opa1, Pink1, Mrpl55, [10,11,17]Ndufs3, [10,11,17]Ndufa2, Glrx5, Diablo, Cpox, Tbrg4, 4930455C21Rik, [10,11,17]Timm8b, Aldh4a1, [10,11,17]Ndufa1, Supv3l1, Ptges2, D9Ertd402e, Mrpl51, Slc25a12, Mecr, Tmem126a, Etfa, [11]Abcb6, [10,11]Ucp3, Nfs1, [11]Abcb8, Acat1, Echs1, Hspe1, [10,11,17]Oxa1l, Tmem143, Lrpprc, Dnaja3, Ecsit, Pet112l, [10,11]Phb, Nt5m, Amacr, [10,11,17]Nnt, [10,11]Phb2, Suclg1, Sdhc, [10,11]Slc25a3, Ndufa11, Afg3l2, [10,11,17]Timm10, Dld, [10,11]Immt, Decr1, Acot2, [10,11]Slc25a11, [10,11,17]Ndufs8, Prdx5, Grpel1, Bckdha, Ghitm, Hspd1, [10,11,17]Ndufa9, Foxred1, [10,11,17]Ndufs4, Ndufa4, [10,11,17]Atp5o, Hint2, [10,11,17]Atp5d, Atp5f1, [10,11]Pmpca, [10,11]Atp5j, Coq7, [10,11]Vdac1, Sucla2, Cs, Bcat2, Hadh, Mrpl12, Acadm, Hspa9, Sod2, Acads, [10,11,17]Rhot2, Slc25a4, Idh3b, Mrpl42, [10,11,17]Ndufs7, Gbas, [10,11]Sdhd, Acadvl, [10,11,17]Sdha, Pdk1,

	Pcx, Chchd3, [10,11,17]Atp5g3, [10,11,17]Ndufs1, Pdha1, [10,11]Mtx2, Trak1, Idh2, Slc25a13, [10,11,17]Atp5c1, [10,11,17]Atp5a1, Me3, [10,11,17]Mfn2, [10,11,17]Atp5b, Fh1, [10,11,17]Ndufs2, Ndufa8, Etfb, [10,11]Ogdh, Sdhb, [10,11,17]Ndufv1, Cyc1, [10,11,17]Uqcrc1, Cox7a1, Cisd1, Cpt1b, Aco2, Pdk4, Ckmt2, 8430408G22Rik
**9 KEGG CITRATE CYCLE TCA CYCLE**	Mdh1, Idh2, Ogdhl, Sdhc, Fh1, Sdhd, Cs, Idh3b, Ogdh, Mdh2, Pdha1, Sdhb, Sdha, Dlat, Dlst, Dld, Idh3a, Idh3g, Sucla2, Pdhb, Suclg1, Aco2, Pck1, Pcx, Acly
**12 KEGG ALZHEIMERS DISEASE**	Ndufv3, Itpr3, Ppp3cc, Ndufb3, Ndufa6, Il1b, Ndufb6, Ndufs3, Ndufa2, Ndufa1, Ndufb2, Sdhc, Cox8a, Cox7a2, Ndufc2, Atf6, Cox6c, Ndufs8, Ndufv2, Ndufa7, Ndufb7, Itpr2, Ndufa9, Ndufs4, Ndufa4, Ryr3, Atp5o, Uqcrh, Cox7b, Atp5d, Atp5f1, Atp5j, Ndufb9, Uqcrc2, Cox4i1, Ndufs7, Sdhd, Uqcr10, Sdha, Ndufs6, Ndufa5, Atp5g3, Ndufs1, Ndufc1, Atp5c1, Ndufb8, Atp5a1, Atp5b, Ndufs2, Ndufa3, Atp2a2, Ndufa8, Uqcrfs1, Cox6b1, Sdhb, Ndufb5, Ndufb10, Ndufa10, Ndufv1, Cyc1, Uqcrc1, Cox7a1, Cox6a2, Cox5a, Uqcrq, Lpl
**13 ORGANELLE INNER MEMBRANE**<br>16 MITOCHONDRIAL INNER MEMBRANE	[16]Timm50, [16]Ndufa6, [16]Ndufb6, [16]Hccs, [16]Alas2, [16]Ndufa13, [16]Opa1, [16]Ndufs3, [16]Ndufa2, Narf, [16]Timm8b, [16]Ndufa1, [16]Ucp3, [16]Oxa1l, [16]Phb, [16]Nnt, [16]Phb2, [16]Slc25a3, [16]Timm10, [16]Immt, [16]Slc25a11, [16]Ndufs8, [16]Ndufa9, [16]Ndufs4, [16]Atp5o, [16]Atp5d, [16]Pmpca, [16]Atp5j, [16]Ndufs7, [16]Sdhd, [16]Sdha, [16]Atp5g3, [16]Ndufs1, [16]Atp5c1, [16]Atp5a1, [16]Atp5b, [16]Ndufs2, [16]Ndufv1, [16]Uqcrc1
**14 KEGG HUNTINGTONS DISEASE**	Ndufb3, Ndufa6, Ndufb6, Vdac2, Ndufs3, Ndufa2, Ucp1, Ndufa1, Polr2e, Ndufb2, Sdhc, Cox8a, Cox7a2, Ndufc2, Cox6c, Ndufs8, Ndufv2, Pparg, Ndufa7, Ndufb7, Ndufa9, Ndufs4, Ndufa4, Atp5o, Uqcrh, Cox7b, Atp5d, Atp5f1, Atp5j, Vdac1, Ndufb9, Sod2, Uqcrc2, Cox4i1, Slc25a4, Ndufs7, Sdhd, Uqcr10, Sdha, Ndufs6, Ndufa5, Atp5g3, Ndufs1, Ndufc1, Atp5c1, Ndufb8, Atp5a1, Atp5b, Ndufs2, Ndufa3, Ndufa8, Uqcrfs1, Cox6b1, Sdhb, Ndufb5, Ndufb10, Ndufa10, Ndufv1, Cyc1, Ppargc1a, Uqcrc1, Cox7a1, Cox6a2, Cox5a, Uqcrq
**15 REACTOME PYRUVATE METABOLISM AND TCA CYCLE**<br>18 REACTOME CITRIC ACID CYCLE	Bsg, [18]Nnt, [18]Suclg1, [18]Sdhc, Pdhx, [18]Dld, [18]Dlst, [18]Sucla2, Pdk2, [18]Cs, Ldha, [18]Idh3g, Ldhb, [18]Idh3b, [18]Sdhd, [18]Sdha, Pdk1, Pdha1, Dlat, [18]Idh2, Pdhb, [18]Idh3a, [18]Fh1, [18]Ogdh, [18]Sdhb, [18]Aco2, [18]Mdh2, Pdk4, Slc16a1
**19 KEGG CARDIAC MUSCLE CONTRACTION**	Cox8a, Cox7a2, Cox6c, Atp1a1, Uqcrh, Cox7b, Tnnt2, Uqcrc2, Cox4i1, Uqcr10, Tnni3, Myl2, Actc1, Atp1b1, Atp2a2, Myh6, Uqcrfs1, Cox6b1, Ryr2, Cacna2d2, Cyc1, Uqcrc1, Cox7a1, Myl3, Cox6a2, Tnnc1, Cox5a, Uqcrq, Myh7
**20 REACTOME DIABETES PATHWAYS**<br>1 REACTOME GLUCOSE REGULATION OF INSULIN SECRETION<br>3 REACTOME ELECTRON TRANSPORT CHAIN<br>6 REACTOME REGULATION OF INSULIN SECRETION	Dnajb11, [1,6]Ndufv3, [1,6]Snap25, [6]Itpr3, Hspa5, [1,6]Gpi1, Pappa, [1,6]Pfkfb3, [1,3,6]Ndufb3, [6]Gngt2, [1,6]Pfkfb1, [1,3,6]Ndufa6, [6]Gng2, [1,3,6]Ndufb6, [6]Glp1r, [1,3,6]Ndufa13, Pla2g7, Dnajb9, [1,3,6]Ndufs3, [1,3,6]Ndufa2, [1,6]Eno1, [1,6]Tpi1, Igf2, [1,6]Pkm2, [1,6]Gck, [6]Gng11, [1,3,6]Ndufa1, [1,3,6]Etfa, [1,3,6]Ndufb2, [1,6]Rapgef3, [1,3,6]Ndufa12, [1,6]Pfkl, [1,6]Nnt, [1,6]Suclg1, [1,3,6]Sdhc, [1,3,6]Cox8a, [1,6]Pdhx, [1,3,6]Ndufa11, [1,6]Pfkfb2, [1,3,6]Ndufb11, [1,3,6]Ndufc2, [1,6]Abcc8, Atf6, [1,6]Dld, [1,3,6]Cox6c, [1,3,6]Ndufs8, [1,3,6]Ndufv2, Herpud1, [1,3,6]Ndufa7, [1,3,6]Ndufb7, [6]Itpr2, [1,6]Atp5k, [6]Prkar2b, [1,3,6]Ndufa9, [1,3,6]Ndufs4, Igfbp3, [1,3,6]Ndufa4, [1,6]Atp5o, [1,6]Dlst, [1,3,6]Cox7b, [1,6]Atp5j2, [1,6]Atp5d, [1,6]Atp5f1, [1,6]Atp5j, [1,6]Aldoa, [1,6]Kcnj11, [1,3,6]Ndufb9, [1,6]Sucla2, [1,6]Cs, [1,3,6]Uqcrc2, [1,6]Idh3g, [1,3,6]Cox4i1, [1,6]Slc25a4, [1,6]Idh3b, [1,3,6]Ndufs7, [1,3,6]Etfdh, [1,3,6]Sdhd, [1,3,6]Uqcr10, [1,3,6]Sdha, [1,3,6]Ndufs6, [1,3,6]Ndufa5, [1,6]Rapgef4, [1,6]Pcx, [1,3,6]Ndufs1, [1,6]Pdha1, [1,6]Dlat, [1,6]Idh2, [1,3,6]Ndufc1, [1,6]Atp5c1, [1,6]Pdhb, [1,3,6]Ndufb8, [1,6]Idh3a, [1,6]Atp5a1, [1,6]Atp5b, [1,6]Fh1, [1,6]Pfkm, [1,3,6]Ndufs2, [1,3,6]Ndufa3, [1,3,6]Ndufa8, [1,3,6]Etfb, [1,3,6]Uqcrfs1, [1,3,6]Cox6b1, [1,6]Ogdh, [1,3,6]Sdhb, [1,3,6]Ndufb5, [1,3,6]Ndufb10, [1,3,6]Ndufa10, [1,6]Cacna2d2, [1,6]Pgam2, [1,3,6]Ndufv1, [1,3,6]Cyc1, [1,3,6]Uqcrc1, [1,6]Eno3, [1,6]Aco2, [1,6]Mdh2, [1,3,6]Cox5a, [1,3,6]Uqcrq, [6]Kcng2
**21 REACTOME GLUCOSE METABOLISM**	Gpi1, Pfkfb3, Pfkfb1, Ugp2, Fbp2, Eno1, Tpi1, Pkm2, Slc25a12, Pgm2, Pygb, Gbe1, Pfkl, Gyg, Pfkfb2, Slc25a11, Agl, Pygl, Pck1, Gys1, Aldoa, Gys2, Pcx, Slc25a13, Pygm, Pfkm, Mdh1, Pgam2, Eno3, Mdh2, Got1
**22 ENERGY DERIVATION BY OXIDATION OF ORGANIC COMPOUNDS**	Gck, Gfpt2, Adrb3, Pygb, [23]Oxa1l, [23]Nnt, [23]Sdhc, [23]Ndufs4, [23]Uqcrc2, [23]Sdhd, Crat, Acadvl, Gys2, [23]Sdha, [23]Ndufs1, Pygm, [23]Pdhb, [23]Me3, [23]Sdhb, [23]Ppargc1a, [23]Uqcrc1, [23]Aco2

23 CELLULAR RESPIRATION	
**24 REGULATION OF HEART CONTRACTION**	Kcnj12, Kcne1, Tnnt2, Kcnq1, Hspb7, Actc1, Chrm2, Cacna1h, Myh6, Ryr2, Scn5a, Kcng2, Des, Myh7
**25 KEGG PPAR SIGNALING PATHWAY**	Angptl4, Dbi, Acsl5, Acsl6, Ucp1, Fabp7, Fabp4, Slc27a1, Scp2, Plin1, Pparg, Scd1, Acsl1, Acox1, Pck1, Adipoq, Acadm, Ppara, Slc27a2, Aqp7, Cpt2, Nr1h3, Rxrg, Acadl, Cpt1b, Sorbs1, Cd36, Lpl

The top 25 *P. gingivalis* gene sets whose member genes are predominantly downregulated with respect to the chronic control group. Column 1 includes the gene set name (bold) as provided by MSigDB and the rank of the gene set based on Normalized Enrichment Score (NES). Gene set names that are not in bold are gene sets that are redundant or partially redundant and included within larger bolded gene sets. The NES ranged from -3.24 (for REACTOME Integration of Energy Metabolism, the top ranked gene set) to -2.33 (KEGG PPAR Signaling Pathway, the 25th-ranked gene set). FDR $q < 1 \times 10^{-30}$. Column 2 includes those genes within the gene set that significantly contributed to the enrichment of the gene set. Genes within redundant gene sets are preceded by corresponding superscripts for the gene set to which they belong.

Supplemental Table 4 - Gene Set Enrichment Analysis. Positive enrichment: chronic *C. pneumoniae*-treated group vs. chronic control group

Gene Set	Genes Contributing to Core Enrichment
**1 MICROBODY**<br>**2 PEROXISOME**<br>20 PEROXISOME ORGANIZATION AND BIOGENESIS<br>7 KEGG PROPANOATE METABOLISM	Gnpat, Pipox, Peci, Amacr, Ide, Ech1, Crot, [20]Pex12, Acot8, [20]Pex1, [20]Pex7, Acot4, Acox3, [20]Pex19, Pnpla8, Scp2, Acad11, Lonp2, [7]Mlycd, [20]Pex14, [20]Pex6, Pex11a, Hsd17b4, Pmvk, Slc25a17, Pxmp4, [20]Pex3, [20]Pex13, [20]Abcd3, [20]Pex16, Hacl1, Abcd2, Ehhadh, Slc27a2
**3 KEGG PEROXISOME**<br>12 REACTOME PEROXISOMAL LIPID METABOLISM	Pipox, [12]Acaa1a, Peci, [12]Amacr, Ech1, [12]Crot, Ddo, Pex12, [12]Acot8, [12]Abcd1, Sod1, Pex1, Pex7, [12]Acox3,Ephx2, Pxmp3, Pex19, Mpv17l, Abcd4, [12]Crat, [12]Scp2, Pex5, Mlycd, Acsl1, Pex14, Acsl3, [12]Acox1, Pex6, Pex11a, Pecr, [12]Hsd17b4, Nudt12, Decr2, Pmvk, [12]Slc25a17, Pxmp4, Pex3, Pex13, [12]Idh1, Dhrs4, Cat, Abcd3, Pex16, [12]Hacl1, Abcd2, Ehhadh,[12]Slc27a2, Acsl5; (Gnpat – 12 ONLY)
**4 KEGG PPAR SIGNALING PATHWAY**	Apoc3, Fabp5, Cyp27a1, Acox3, Rxra, Acadm, Slc27a1, Acadl, Scp2, Gyk, Acsl1, Cpt2, Acsl3, Acox1, Nr1h3, Ppara, Dbi, Angptl4, Aqp7, Pparg, Scd1, Fabp4, Ehhadh, Slc27a2, Me1, Ucp1, Acsl5, Plin1, Adipoq, Pck1
**5 REACTOME METABOLISM OF LIPIDS AND LIPOPROTEINS**<br>6 REACTOME REGULATION OF LIPID METABOLISM BY PEROXISOME PROLIFERATOR ACTIVATED RECEPTOR ALPHA<br>22 REACTOME CHOLESTEROL BIOSYNTHESIS<br>23 REACTOME SYNTHESIS OF BILE ACIDS AND BILE SALTS VIA 7ALPHA HYDROXYCHOLEST EROL	Crot, Cav1, Cyp46a1, Ldlr, Acat1, [23]Acot8, Abcd1, Apoc3, Kdsr, Tm7sf2, Scarb1, Prkaca, Hsd11b1, Echs1, [23]Cyp27a1, Hadha, Acox3, Sptlc1, [6]Abca1, [6]Rxra, [6]Acadm, [6]Slc27a1, Crat, Stard5, Abhd5, Acadl, Decr1, [6,23]Scp2, Gyk, Hadh, Prkd1, [22]Cyp51, [23]Hsd3b7, [6]Acsl1, Dci, [6]Cpt2, Acads, Agpat1, [6]Acox1, Sgpl1, Gpam, [6]Nr1h3, [6]Ppara, Lass2, [6]Pex11a, [23]Hsd17b4, [22]Dhcr7, Acadvl, [22]Pmvk, [6]Angptl4, Slc25a17, [22]Sqle, Agpat3, [22]Sc5d, [22]Fdft1, Idh1, [6]Srebf1, Slc25a20, [22]Lss, Acacb, [6]Plin2, [6]Scd1, Hacl1, [6]Fabp4, [6]Ehhadh, [6]Dgat1, [22]Dhcr24, [23]Slc27a2, [6]Me1, [6]Ucp1, Lipe, Agpat2, [6]Plin1, Mgll, [22]Nsdhl, [6]Acaca, Gpd1, [6]Fasn, Gpd2; (Amacr – 23 ONLY)
**8 KEGG VALINE LEUCINE AND ISOLEUCINE DEGRADATION**<br>7 KEGG PROPANOATE METABOLISM	[7]Acat1, [7]Mcee, [7]Echs1, Hibadh, [7]Hadha, Aox1, Bcat2, Acaa2, [7]Acadm, Dld, Mccc1, Acad8, Acadsb, [7]Mut, Hadh, Bckdhb, Acads, Mccc2, [7]Aldh6a1, [7]Hibch, [7]Aldh2, [7]Aldh9a1, Bckdha, [7]Pccb, Dbt, [7]Pcca, [7]Ehhadh, [7]Acat2
**9 KEGG FATTY ACID METABOLISM**	Aldh3a2, Acaa1a, Peci, Adh5, Acat1, Echs1, Hadha, Acox3, Acaa2, Acadm, Acadsb, Acadl, Hadh, Adh7, Acsl1, Dci, Cpt2, Acads, Acsl3, Acox1, Acadvl, Aldh2, Aldh9a1, Ehhadh, Adh1, Acat2, Acsl5
**10 REACTOME METABOLISM OF VITAMINS AND COFACTORS**	Pank2, Nadsyn1, Aasdhppt, Rfk, Tpk1, Mthfd1, Ppcdc, Flad1, Shmt1, Slc25a16, Cyb5, Gsto2, Slc5a6, Slc25a32, Slc46a1, Nadk, Slc19a1, Coasy, Pank3, Pank1, Acp5, Fasn

**11 KEGG PYRUVATE METABOLISM**<br>7 KEGG PROPANOATE METABOLISM	Acat3, Haghl, [7]Ldha, Hagh, Dlat, Acat1, Acyp1, Pdha1, Pdhb, Dld, Pkm2, Grhpr, Ldhd, Aldh2, Aldh9a1, [7]Acacb, Me1, Pcx, [7]Acss2, [7]Acaca, Pck1, Acat2
**13 KEGG GLYCEROLIPID METABOLISM**	Agpat9, Dgkb, Gyk, Agk, Agpat6, Agpat1, Gpam, Aldh2, Agpat3, Aldh9a1, Dgat2, Dgat1, Pnpla3, Agpat2, Mgll
**15 LIPID CATABOLIC PROCESS**<br>24 CELLULAR LIPID CATABOLIC PROCESS	[24]Smpd3, [24]Prdx6, [24]Aldh5a1, [24]Ech1, [24]Cyp46a1, [24]Pla2g4c, Angptl3, [24]Echs1, [24]Acadm, [24]Acads, [24]Ppara, [24]Acadvl, [24]Hacl1, [24]Pnpla3, Cidea
**16 MITOCHONDRION**<br>14 MITOCHONDRIAL LUMEN<br>17 MITOCHONDRIAL MATRIX<br>7 KEGG PROPANOATE METABOLISM	Timm8b, Spg7, Prelid1, Trnt1, Polrmt, Car5a, Tomm22, Immt, Asah2, Caprin2, Hscb, Fdxr, Casp7, Dguok, Tmem186, Opa1, Ccdc39, Slc9a6, Cpt1b, Abcb6, Sdhb, Dnajc19, Atp5d, Ndufa1, Cisd1, Sco1, Gpx4, Fdx1, Tsfm, Hint2, Mrpl32, Mtx2, Supv3l1, Fh1, Gcdh, Hsd3b1, Uqcrc1, Cox15, Cln3, Agpat5, Napg, [14,17]Polg2, Sod2, Tbrg4, [14,17]Mrpl40, [14,17]Timm44, Ndufb6, [14,17]Mrps24, Hspe1, Hspd1, Phb2, Cyc1, Sdhd, Mthfd1, Hsd17b10, Mtrf1, Peci, Afg3l2, Ndufs4, Mrps31, Glrx2, [14,17]Mrps15, Surf1, Aldh5a1, Amacr, [14,17]Cs, Idh3b, Slc25a15, Coq4, [14,17]Etfa, Ppox, [14,17]Mrps35, [14,17]Mrps18a, Pet112l, Ndufs3, [14,17]Etfb, Maob, D2hgdh, Ndufs8, Clpx, [14,17]Mrps11, [14,17]Dnaja3, Cox4nb, Slc22a4, Hspa9, Acat1, Tufm, Sdha, Oxsm, Sod1, Timm9, Pdha1, [14,17]Mrps22, Htra2, Mcat, [14,17]Nr3c1, Echs1, [14,17]Bckdk, Mosc2, Foxred1, Timm50, Ecsit, Cox11, Acn9, Mtif3, Thg1l, Ndufs5, Bcs1l, Casp8, Bcat2, Ptges2, Alas2, Glud1, [14,17]Acadm, Dld, Smcr7l, [14,17]Aldh4a1, Acp6, Mtif2, Acadsb, Aco2, Tfam, Abcb8, Tmem143, Ghitm, Aifm1, Aass, Glrx5, Mut, Decr1, Lonp1, Triap1, [7]Sucla2, Hadh, Bphl, Phb, Slc25a22, [14,17]Mrpl10, [7]Suclg1, Sars2, Bnip3l, Iscu, [14,17]Bckdhb, Txnrd2, Acads, Ak3, Gcsh, Cox18, [14,17]Grpel1, Ldhd, Pts, Acadvl, [14,17]Mrps21, Dhrs4, [14,17]Bckdha, Pccb, [14,17]Gm4459, Ucp3, Coq6, Pdk4, [14,17]Dbt, Aifm2, Gstz1, Letm1, Car5b, Nipsnap1, Mecr, Pcx, Slc25a1
**18 REACTOME BRANCHED CHAIN AMINO ACID CATABOLISM**	Auh, Hsd17b10, Acat1, Hibadh, Bcat2, Dld, Mccc1, Acad8, Acadsb, Bckdhb, Mccc2, Aldh6a1, Hibch, Bckdha, Dbt
**19 COFACTOR METABOLIC PROCESS**	Sdhb, Mocos, Fech, Acot3, Cox15, Gclc, Blvra, Gclm, Sdhd, Pdss2, Glrx2, Ppox, Alas1, Sdha, Oxsm, Sod1, Pgls, Acot4, Pdhb, Alas2, Aco2, Gpx1, Tspo, Alad, Mlycd, Iscu, Mocs2, Coasy Aldh1l1, Me1, Pgd, Acly
**21 KEGG BIOSYNTHESIS OF UNSATURATED FATTY ACIDS**	Acot4, Hadha, Acox3, Acox1, Pecr, Elovl5, Hsd17b12, Scd1, Ptplb, Elovl6
**25 VITAMIN METABOLIC PROCESS**	Pgls, Rxra, Dhrs3, Retsat, Dhrs9, Me1, Pgd

The top 25 *C. pneumoniae* gene sets whose member genes are predominantly upregulated with respect to the chronic control group. Column 1 includes the gene set name (bold) as provided by MSigDB and the rank of the gene set based on Normalized Enrichment Score (NES). Gene set names that are not in bold are gene sets that are redundant or partially redundant and included within larger bolded gene sets. The NES ranged from 2.64 (for Microbody Peroxisome, the top ranked gene set) to 2.03 (Vitamin Metabolic Process, the 25th-ranked gene set). FDR *q* range: 0-0.001. Column 2 includes those genes within the gene set that significantly contributed to the enrichment of the gene set. Genes within redundant gene sets are preceded by corresponding superscripts for the gene set to which they belong.

Supplemental Table 5 - Gene Set Enrichment Analysis. Negative enrichment: chronic *C. pneumoniae*-treated group vs. chronic control group

Gene Set	Genes Contributing to Core Enrichment
**1 KEGG HYPERTROPHIC CARDIOMYOPATHY**	Cacna1d, Itga5, Itgb8, Tgfb1, Prkag3, Itga2, Cacna2d1, Sgcb, Cacnb2, Lama2, Prkaa2, Sgcg, Itgb6, Sgca, Slc8a1, Cacna1c, Atp2a2, Ryr2, Cacna2d2, Des, Actc1, Myh7, Tnni3, Myh6, Myl2, Myl3, Tnnc1, Ttn, Mybpc3, Tnnt2
**2 KEGG DILATED CARDIOMYOPATHY**	Prkx, Itga2, Cacna2d1, Sgcb, Cacnb2, Lama2, Sgcg, Itgb6, Sgca, Slc8a1, Cacna1c, Atp2a2, Ryr2, Cacna2d2, Des, Actc1, Myh7, Tnni3, Myh6, Myl2, Myl3, Tnnc1, Ttn, Mybpc3, Tnnt2
**3 REACTOME MUSCLE CONTRACTION**<br>4 REACTOME STRIATED MUSCLE CONTRACTION	[4]Mybpc2, 2900073G15Rik, [4]Ttn, [4]Tnnt2, [4]Tnni3, [4]Tnni1, [4]Tnnc1, [4]Tmod1, [4]Tcap, Sorbs1, [4]Neb, [4]Myl2, [4]Myl3, [4]Myl4, [4]Myh6, [4]Mybpc3, [4]Des, [4]Actn2
**5 MUSCLE DEVELOPMENT**<br>21 SKELETAL MUSCLE DEVELOPMENT	Gaa, Tgfb1, Hbegf, [21]Svil, [21]Notch1, Sgcb, [21]Tbx3, Ache, Mstn, [21]Nrd1, [21]Cacnb2, Lama2, Sgcg, Sgca, [21]Mapk12, [21]Igfbp3, [21]Cacna1h, Itgb1bp2, [21]Acta1, Myh7, Tnni3, Myh6, [21]Ttn, Mybpc3, [21]Csrp3
**6 ACTIN CYTOSKELETON**	Fscn1, Gsn, Ablim1, Sep11, Capg, Pdlim5, Spna1, Amot, Sorbs1, Neb, Clic5, Dynll2, Myom1, 2900073G15Rik, Ablim2, Spnb1, Myo18b, Palld, Acta1, Myl4, Abra, Actc1, Myh7, Tnni3, Myh6, Myl3, Tnnc1, Actn2, Tnnt2, Myot
**8 STRUCTURAL MOLECULE ACTIVITY**<br>7 STRUCTURAL CONSTITUENT OF MUSCLE	Fbn2, Actl7b, Mybpc1, Strn4, Mtap7, Mtap4, Plp1, Mal, Epb4.1l4b, Spna1, Lama2, Lad1, [7]Neb, Fbln2, [7]Myom1, Comp, Chi3l1, [7]2900073G15Rik, Fbln1, Dsp, Myoc, [7]Tcap, [7]Mybpc2, Acta1, Des, [7]Actc1, [7]Myh7, [7]Myom2, [7]Myh6, [7]Myl2, [7]Myl3, [7]Ttn, [7]Actn2, [7]Mybpc3, [7]Myot
**9 REGULATION OF MULTICELLULAR ORGANISMAL PROCESS**<br>12 REGULATION OF HEART CONTRACTION<br>25 REGULATION OF MUSCLE CONTRACTION	Grik2, [12]Kcnj12, [25]Sphk1, Tlr8, Icosl, [25]2900073G15Rik, [12]Kcnq1, [12]Kcne1, [12]Scn5a, [25]Ppp1r12b, Ptgds, [12]Cacna1h, [12]Ryr2, [12]Hspb7, [12]Kcng2, [12]Chrm2, Ahsg, [12]Des, [12]Actc1, [12]Myh7, [12]Myh6, [25]Myl2, [25]Tnnc1, [25]Mybpc3, [12]Tnnt2
**10 KEGG CARDIAC MUSCLE CONTRACTION**	Slc8a1, Cacna1c, Atp1a1, Atp2a2, Ryr2, Cacna2d2, Atp1b1, Actc1, Myh7, Tnni3, Myh6, Myl2, Myl3, Tnnc1, Cox6a2, Tnnt2
**11 CYTOSKELETAL PROTEIN BINDING**<br>23 ACTIN BINDING	Myo9b, Tnnt1, Spnb2, Prnp, Ccr5, Rab11fip5, Apoe, Ezr, Kif5b, Mybpc1, Arfgef1, Baiap2, [23]Svil, [23]Ablim1, Ctnna1, Pacsin3, [23]Egfr, [23]Pdlim5, Tmod4, [23]Shroom3, [23]Spna1, [23]Ssh2, Jup, [23]Sorbs1, [23]Scin, [23]Fxyd5, Clip1, [23]Nexn, Clasp1, [23]Twf2, [23]Spnb1, Trim63, Palld, Acta1, [23]Abra, [23]Tnni3, [23]Nrap, Tnnc1, Ttn, Actn2, Mybpc3, [23]Tnnt2
**13 KEGG ARRHYTHMOGENIC RIGHT VENTRICULAR CARDIOMYOPATHY ARVC**	Cacna1d, Itga5, Itgb8, Itga2, Cacna2d1, Sgcb, Ctnna1, Ctnna3, Cacnb2, Lama2, Jup, Dsc2, Sgcg, Itgb6, Dsg2, Sgca, Slc8a1, Cacna1c, Dsp, Atp2a2, Pkp2, Ryr2, Cacna2d2, Cdh2, Des, Actn2
**14 CONTRACTILE FIBER**<br>**15 CONTRACTILE FIBER PART**<br>19 MYOFIBRIL	[19]Neb, [19]Myom1, 2900073G15Rik, [19]Acta1, [19]Abra, [19]Des, [19]Myoz2, [19]Tnni3, Myl3, [19]Tnnc1, [19]Ttn, [19]Tnnt2

**16 HEART DEVELOPMENT**	Irx4, Fgf12, Gata4, Tbx5, Hand2, Alpk3, Casq2, Myh7, Myh6, Mybpc3
**20 GATED CHANNEL ACTIVITY**<br>17 VOLTAGE GATED CHANNEL ACTIVITY<br>18 VOLTAGE GATED CATION CHANNEL ACTIVITY	Trpc4, Kcnmb4, Chrnb4, [17,18]Kcnd3, [17,18]Cacna1d, Htr3a, [17,18]Kcnk1, [17,18]Kcnk6, Nmur2, [17,18]Kcnh2, [17,18]Kcnb2, Clcn1, [17,18]Kcnj10, [17,18]Kcna5, [17,18]Cacna2d1, Trpc3, Chrna3, [17,18]Kcna6, [17,18]Scn4b, [17,18]Kcna1, [17,18]Scn7a, [17,18]Cacnb2, [17,18]Kcnj12, [17,18]Kcna4, [17,18]Scn4a, Ryr3, [17,18]Kcnq1, [17,18]Kcne1, Kcnn2, [17,18]Cacna1c, [17,18]Scn5a, [17,18]Cacna1g, [17,18]Kcnj5, [17,18]Cacna1h, [17,18]Kcnj3, Ryr2, [17,18]Kcng2
**22 CATION CHANNEL ACTIVITY**	Kcns1, Trpv5, Trpc4, Kcnmb4, Chrnb4, Kcnd3, Cacna1d, Htr3a, Kcnk1, Kcnk6, Kcnh2, Kcnb2, Kcnj10, Kcna5, Cacna2d1, Trpc3, Chrna3, Kcna6, Scn4b, Kcna1, Scn7a, Cacnb2, Kcnj12, Kcna4, Scn4a, Ryr3, Kcnq1, Kcne1, Kcnn2, Cacna1c, Scn5a, Cacna1g, Kcnj5, Cacna1h, Kcnj3, Ryr2, Kcng2
**24 CYTOSKELETAL PART**	Nek2, Klc1, Myo9b, Gas2, Spnb2, Cenpf, Nefl, Dnaic2, Nedd9, Shroom4, Ezr, Kif5b, Mtap7, Kifc3, Mtap4, Sept11, Capg, Spna1, Amot, Drd2, Narf, Sorbs1, Dynll2, Clip1, Myom1, 2900073G15Rik, Cacna1c, Clasp1, Spnb1, Myo18b, Palld, Acta1, Myl4, Actc1, Myh7, Tnni3, Myh6, Myl3, Tnnc1, Actn2, Tnnt2

The top 25 *C. pneumoniae* gene sets whose member genes are predominantly downregulated with respect to the chronic control group. Column 1 includes the gene set name (bold) as provided by MSigDB and the rank of the gene set based on Normalized Enrichment Score (NES). Gene set names that are not in bold are gene sets that are redundant or partially redundant and included within larger bolded gene sets. The NES ranged from -2.79 (for KEGG Hypertrophic Cardiomyopathy, the top ranked gene set) to -2.23 (Regulation of Muscle Contraction, the 25th-ranked gene set). FDR $q < 1 \times 10^{-30}$. Column 2 includes those genes within the gene set that significantly contributed to the enrichment of the gene set. Genes within redundant gene sets are preceded by corresponding superscripts for the gene set to which they belong.

Supplemental Table 6 - Gene Set Enrichment Analysis. Positive enrichment: Western diet group vs. chronic control group

Gene Set	Genes Contributing to Core Enrichment
**1 KEGG LYSOSOME**	Arsb, Ap3s2, Sumf1, Mcoln1, Ap1g1, Scarb2, Galns, Lamp1, Ap3b1, Nagpa, Gaa, Atp6v0b, Atp6v0a2, M6pr, Ctns, Naglu, Atp6v1h, Atp6ap1, Hgsnat, Naga, Ppt1, Ap1s2, Ap3m2, Ctsw, Cd63, Atp6v0a1, Tpp1, Atp6v0d1, Aga, Npc1, Gm2a, Lamp2, Ap1b1, Gba, Ap1s1, Slc17a5, Clta, Gnptab, Gns, Neu1, Ctsa, Acp2, Fuca1, Pla2g15, Psap, Ctsh, Igf2r, Acp5, Tcirg1, Asah1, Ctsk, Hexa, Cln3, Glb1, Hexb, Dnase2a, Manba, Ctsl, Man2b1, Ctso, Ctsb, Gla, Ctsd, Ctsc, Ctsz, Lgmn, Cln5, Galc, Slc11a1, Lipa, Npc2, Gusb, Atp6v0d2, Laptm5, Cd68, Ctss
**2 REACTOME SIGNALING IN IMMUNE SYSTEM**<br>10 REACTOME INNATE IMMUNITY SIGNALING<br>24 CELL SURFACE INTERACTIONS AT THE VASCULAR WALL	C920025E04Rik, [10]Nfkbib, Ppp2r5c, Map3k8, Angpt2, [10]Map2k4, Fyn, [10]Map2k6, Csk, Gas6, [10]Mapk14, [10]Ticam2, [10]Tlr6, [10]Ikbkg, [10]Ripk3, [10]Ikbke, [10]Mapkapk3, Itk, Jam2, Atp1b3, [10]Rps6ka2, [10]Tlr4, H2-Ob, Icosl, [10]Dusp7, Cd40, [24]Esam, Icam2, [10]Map3k1, B2m, [24]Cd244, [24]F11r, [10]Rps6ka3, [24]Fn1, [24]Slc3a2, Cd28, Cd34, Cd3e, [10]Map2k1, Icam1, [24]Kras, [24]Lck, [24]Itgal, Cd274, [10]Myd88, [24]Nras, [24]Slc7a8, [10]C2, Cd200r1, [24]Slc16a3, Pak1, Ripk2, Grap2, [24]Slc7a7, [10]Mapk3, [24]Pf4, [10]Irak4, Tnfrsf14, H2-M3, [24]Pecam1, [24]Itga6, Cd8b1, [24]Ptpn6, [10]Irak3, [24]Cd47, [24]Olr1, [24]Pros1, Cd3g, [10]Tlr9, [10]Tlr2, [24]Tek, [24]Slc7a11, [10]Dusp6, [10]Irf7, [10]Tlr3, Was, [10]Rps6ka1, Cd4, [10]Ly96, Evl, Fyb, Cd86, [24]Lyn, [10]Tlr7, [24]Itgax, H2-Eb1, [24]Selp, [24]Itgam, [24]Fcer1g, Lcp2, [10]Tlr1, [24]Sirpa, Cd80, Fcgr2b, H2-Aa, [10]Cd180, Tyrobp, [10]Tlr8, Fcgr4, [10]C1ra, [24]Inpp5d, [10]Lbp, H2-DMa, [10]C1qc, [10]C1qa, [10]C3, [24]Cd84, [10]C1qb, Vcam1, Vav1, [10]Ly86, [10]Cfb, [10]Cd14, [24]Cd48, [24]Itga4, [24]Itgb2, Ptprc, [24]Selplg, [10]C6
**3 KEGG NATURAL KILLER CELL MEDIATED CYTOTOXICITY**	Grb2, Klrk1, Vav2, Gm10499, Bid, Nfatc3, Vav3, Fyn, Ppp3cc, Nfatc1, Tnf, Pik3r3, Icam2, Ppp3r1, Ifnar1, Cd244, Tnfsf10, Ppp3ca, Map2k1, Icam1, Kras, Lck, Itgal, Nras, Pak1, Mapk3, H2-M3, Pik3cd, Ptpn6, Ifngr1, Ifnar2, Sh2d1b1, Sh3bp2, Pik3cg, Pik3r5, Fcer1g, Prkcb, Lcp2, 2010110P09Rik, Rac2, Tyrobp, Fcgr4, Sykb, Plcg2, H2-T23, Vav1, Cd48, Ptk2b, Itgb2
**4 KEGG SYSTEMIC LUPUS ERYTHEMATOSUS**	Hist1h2bn, Tnf, H2-Ob, Cd40, Hist2h2ab, Cd28, Hist3h2a, C2, Hist2h2ac, Grin2a, Hist1h2bm, Hist1h2bg, H2afz, Hist1h2bb, Fcgr1, Cd86, Hist2h2bb, H2-Eb1, Cd80, Fcgr2b, H2-Aa, Fcgr4, Fcgr3, C1ra, H2-DMa, C1qc, C1qa, C3, C1qb, Hist1h2ak, C6
**6 KEGG B CELL RECEPTOR SIGNALING PATHWAY**	Akt1, Nfkb1, Malt1, Grb2, Vav2, Nfkbib, Nfatc3, Vav3, Ppp3cc, Nfatc1, Cd79b, Ikbkg, Rasgrp3, Pik3r3, Ppp3r1, Ppp3ca, Map2k1, Kras, Nras, Dapp1, Mapk3, Btk, Pik3cd, Ptpn6, Nfkbie, Cd22, Cd72, Lyn, Pik3cg, Blnk, Pik3r5, Prkcb, 2010110P09Rik, Fcgr2b, Rac2, Pik3ap1, Sykb, Inpp5d, Plcg2, Vav1
**7 LEISHMANIA INFECTION**	Nos2, Nfkbib, Mapk14, Jak2, Tlr4, Tnf, H2-Ob, Il1a, Myd88, Jak1, Mapk3, Irak4, Ptpn6, Tgfb1, Stat1, Ifngr1, Ncf1, Tlr2, Fcgr1, Cyba, H2-Eb1, Itgam, Ncf4, Prkcb, H2-Aa, Fcgr4, Fcgr3, H2-DMa, C3, Itga4, Itgb2
**8 KEGG TOLL LIKE RECEPTOR SIGNALING PATHWAY**	Tollip, Map3k8, Map2k4, Map2k6, Mapk14, Ticam2, Tlr6, Ikbkg, Ikbke, Tlr4, Tnf, Cd40, Pik3r3, Ifnar1, Map2k1, Myd88, Mapk3, Irak4, Pik3cd, Stat1, Cxcl10, Ctsk, Ccl5, Ifnar2, Tlr9, Tlr2, Irf7, Tlr3, Ly96, Cd86, Tlr7, Pik3cg, Irf5, Pik3r5, Tlr1, Cd80, Spp1, Tlr8, Lbp, Casp8, Cd14
**9 IMMUNE SYSTEM PROCESS**<br>5 IMMUNE RESPONSE	Cd79b, Ccr9, Ikbkg, Nlrc3, Il15, Cebpg, Lig1, Myh9, Cxcl13, Atp6v0a2, Tlr4, Map4k1, Icosl, [5]Mr1, [5]Fth1, [5]Ccl12, Cdk6, [5]Il18bp, [5]Ets1, [5]Ctsw, [5]Il6st, [5]Tcf7, [5]Prelid1, [5]Cd28, Acvr1b, Cd34, Cd3e, Lck, Lrmp, [5]Psmb10, [5]Il18, [5]Ifitm2, [5]Cd274, [5]Rsad2, [5]Tapbp, [5]Ciita, [5]C2, [5]Cnr2, [5]Bst2, [5]Gbp2, [5]Ccr2, [5]Ccr1, [5]Il27ra, Pf4, [5]Tnfrsf14, Cklf, [5]Gpr65, [5]Il2ra, [5]Tgfb1, Clec7a, [5]Cxcl12, [5]Sema4d, [5]Ccl5, [5]Dpp4, Cd47, [5]Ifitm3, [5]Il4ra, Prex1, [5]Bnip3l, [5]Ptger4, [5]Il10rb, Hcls1, [5]Nfam1, [5]Cd22, [5]C5ar1, [5]Was, [5]Lat2, Cd4, [5]Ctsc, [5]Fcgr1, [5]Fyb, [5]Cd86, Lyn, [5]Tlr7, [5]Rgs1, [5]Aim2, [5]Irf8, [5]Blnk, [5]Cd74, [5]Ncf4, [5]Lcp2, Sfpi1, [5]Fcgr2b, Saa3, [5]Tlr8, [5]Trem2, [5]Fcgr4, Sykb, [5]Arhgdib, [5]Ptafr, [5]Il7r, [5]Igsf6, [5]Ccr5, Dock2, Mafb, [5]Cd83, [5]Cx3cl1, [5]Ctss, [5]Ly86, [5]Bst1, [5]Il2rg, [5]Itgb2, [5]Ptprc, [5]Cxcr4
**11 REACTOME TOLL RECEPTOR CASCADES**	Ppp2ca, Tirap, Tab2, Ripk1, Mapk9, Nfkb1, Pik3c3, Irak1, Ppp2r1a, Nfkbib, Map2k4, Map2k6, Mapk14, Ticam2, Tlr6, Ikbkg, Ripk3, Ikbke, Mapkapk3, Rps6ka2, Tlr4, Dusp7, Map3k1, Rps6ka3, Map2k1, Myd88, Mapk3, Irak4, Irak3, Tlr9, Tlr2, Dusp6,

	Irf7, Tlr3, Rps6ka1, Ly96, Tlr7, Tlr1, Cd180, Tlr8, Lbp, Ly86, Cd14
**13 REACTOME S PHASE**<br>15 REACTOME SYNTHESIS OF DNA	[15]Rpa1, [15]Psma3, [15]Psmd2, [15]Psma2, [15]Psmd9, [15]Prim2, [15]Psmc6, [15]Cdc6, [15]Psme1, [15]Psmb2, [15]Orc5l, [15]Psmb4, Cul1, [15]Psmd13, [15]Mcm5, [15]Orc4l, [15]Psma6, [15]Psmb6, [15]Rfc5, [15]Mcm3, [15]Psmb3, [15]Orc2l, [15]Psme3, [15]Rb1, [15]Orc3l, [15]Pola1, [15]Psma1, Skp1a, [15]Gins1, [15]Rfc1, [15]Psmd5, [15]Mcm4, [15]Lig1, Ccnh, [15]Rfc4, [15]Cdt1, [15]Psma4, [15]Pola2, Cdk4, [15]Gins4, [15]Psmf1, [15]Pold4, [15]Pold3, [15]Rfc2, [15]Psmd8, [15]Mcm2, [15]Psma7, [15]Psmd10, [15]Pole, [15]Psmb10, [15]Dna2, [15]Psmd14, [15]Rfc3, [15]Psme2, Ccnd1, Ccne2, [15]Rpa3, [15]Psmb9, [15]Psmb8, [15]Ccna2, [15]Pole2, [15]Mcm7
**14 KEGG FC GAMMA R MEDIATED PHAGOCYTOSIS**	Pik3r3, Arpc4, Arf6, Map2k1, Pak1, Mapk3, Arpc3, Pik3cd, Arpc5, Marcks, Ncf1, Was, Arpc1b, Hck, Fcgr1, Lyn, Pik3cg, Pik3r5, Prkcb, Fcgr2b, Rac2, Fcgr4, Sykb, Fcgr3, Inpp5d, Plcg2, Dock2, Vav1, Ptprc
**16 REACTOME HOST INTERACTIONS OF HIV FACTORS**	Nup37, Psmd6, Psmc4, Rac1, Psmd12, Psmc1, Kpnb1, Nup210, Arf1, Psmd1, Nup155, Nup54, Psma3, Psmd2, Psma2, Nupl1, Nup205, Psmd9, Psmc6, Aaas, Xpo1, Nup62, Psme1, Psmb2, Psmb4, Psmd13, Psma6, Psmb6, Psmb3, Ap2s1, Cul5, Npm1, Psme3, Seh1l, Ap1g1, Pacs1, Ran, Fyn, Psma1, Tpr, Skp1a, Psmd5, Nup50, Psma4, Ap2m1, Atp6v1h, Ap2b1, Tceb1, Ap1s2, Psmf1, B2m, Psmd8, Ap2a2, Cd28, Psma7, Psmd10, Lck, Psmb10, Ap1b1, Ap1s1, Psmd14, Psme2, Cd8b1, Psmb9, Psmb8, Elmo1, Cd4, Hck, Dock2
**17 REACTOME M G1 TRANSITION**<br>12 DNA REPLICATION PRE INITIATION	Psmb1, Psmc2, Psmb7, Psma5, Psmd6, Psmc4, Psmd12, Psmc1, [12]Mcm6, [12]Psmd1, [12]Psma3, [12]Psmd2, [12]Psma2, [12]Psmd9, [12]Psmc6, [12]Cdc6, [12]Psme1, [12]Psmb2, [12]Orc5l, [12]Psmb4, [12]Psmd13, [12]Mcm5, [12]Orc4l, [12]Psma6, [12]E2f3, [12]Psmb6, [12]Mcm3, [12]Psmb3, [12]Orc2l, [12]Psme3, [12]Orc3l, [12]Psma1, [12]E2f1, [12]Psmd5, [12]E2f2, [12]Mcm4, [12]Cdt1, [12]Psma4, [12]Psmf1, [12]Psmd8, [12]Mcm2, [12]Psma7, [12]Psmd10, [12]Psmb10, [12]Psmd14, [12]Psme2, [12]Psmb9, [12]Psmb8, [12]Mcm7
**18 KEGG T CELL RECEPTOR SIGNALING PATHWAY**	Mapk9, Akt1, Nfkb1, Malt1, Cbl, Grb2, Vav2, Nck1, Nfkbib, Map3k8, Nfatc3, Vav3, Fyn, Ppp3cc, Nfatc1, Mapk14, Ikbkg, Itk, Tnf, Cdk4, Pik3r3, Ppp3r1, Cd28, Cd3e, Ppp3ca, Map2k1, Kras, Lck, Nras Pak1, Grap2, Mapk3, Pik3cd, Cd8b1, Ptpn6, Nfkbie, Cd3g, Tec, Cd4, Pik3cg, Pik3r5, Lcp2, 2010110P09Rik, Rasgrp1, Vav1, Ptprc
**19 DEFENSE RESPONSE**	Cd40, Gata3, Clec1b, Irak2, F11r, Kng2, Hp, Tnfaip6, Nmi, Il1a, Nlrc4, Cd5l, Rsad2, Lsp1, C2, Tnfrsf1a, Rnase6, Ripk2, Cbara1, Anxa1, Ccr2, Ccr1, Stab1, Tnip1, H2-M3, Nlrp3, Tgfb1, Sele, Tcirg1, Cxcl10, Ccl5, Ncf1, Bnip3l, Il10rb, C5ar1, Tlr3, Was, Cysltr1, Aif1, Ly96, Nod1, Clec5a, Cx3cr1, Tlr7, Ccl3, Cfp, Blnk, Il1rl2, Csf3r, Ccl2, Tyrobp, Tlr8, Aoah, Ptafr, Lbp, Alox5ap, Ccr5, Cd84, Cd83, C3ar1, Cx3cl1, Cd48, Kcnn4, Lgals3bp, Ptprc, Cxcr4, Pla2g7, Cybb
**20 KEGG NOD LIKE RECEPTOR SIGNALING PATHWAY**	Hsp90b1, Nfkbib, Mapk14, Ikbkg, Tnf, Ccl12, Sugt1, Erbb2ip, Xiap, Birc3, Card9, Nlrc4, Il18, Card6, Ripk2, Casp1, Mapk3, Ccl7, Nlrp3, Pycard, Ccl5, Nod1, Pstpip1, Ccl2, Tnfaip3, Casp8
**21 KEGG HEMATOPOIETIC CELL LINEAGE**	Tnf, Cd5, Kit, Cd34, Cd3e, Il1a, Il2ra, Itga6, Cd8b1, Cd44, Il4ra, Cd3g, Cd22, Csf2ra, Cd4, Tfrc, Fcgr1, Cd38, H2-Eb1, Itgam, Csf3r, Anpep, Csf1r, Il7r, Cd14, Cd37, Itga4
**22 REACTOME G1 S TRANSITION**<br>12 DNA REPLICATION PRE INITIATION	[12]Rpa1, [12]Psma3, [12]Psmd2, [12]Psma2, [12]Psmd9, [12]Prim2, [12]Psmc6, Fbxo5, [12]Cdc6, [12]Psme1, [12]Psmb2, [12]Orc5l, [12]Psmb4, Cul1, [12]Psmd13, [12]Mcm5, [12]Orc4l, [12]Psma6, [12]E2f3, [12]Psmb6, [12]Mcm3, [12]Psmb3, [12]Orc2l, [12]Psme3, Rb1, [12]Orc3l, [12]Pola1, [12]Psma1, Skp1a, [12]E2f1, [12]Psmd5, [12]E2f2, [12]Mcm4, Ccnh, [12]Cdt1, [12]Psma4, [12]Pola2, Tyms, [12]Psmf1, [12]Psmd8, [12]Mcm2, [12]Psma7, [12]Psmd10, [12]Pole, [12]Psmb10, Tk2, [12]Psmd14, [12]Psme2, Ccne2, [12]Rpa3, [12]Psmb9, [12]Psmb8, Ccna2, [12]Dbf4, [12]Pole2, [12]Mcm7, Cdk1, Rrm2
**23 KEGG CHEMOKINE SIGNALING PATHWAY**	Ccr7, Gnb1, Grb2, Ccl22, Stat5b, Vav2, Gnai2, Nfkbib, Vav3, Adrbk1, Stat2, Cxcl5, Csk, Ccr9, Jak2, Ikbkg, Adcy4, Itk, Cxcl13, Jak3, Rap1a, Pik3r3, Cxcr6, Ccl12, Pxn, Gng11, Map2k1, Kras, Prkx, Gnai3, Nras, Pak1, Ccr2, Mapk3, Ccr1, Pf4, Gng2, Pik3cd, Ccl7, Stat1, Cxcl12, Cxcl10, Rap1b, Ccl5, Elmo1, Fgr, Prex1, Ncf1, Was, Hck, Arrb2, Cx3cr1, Arrb1, Lyn, Plcb2, Ccl3, Pik3cg, Pik3r5, Gngt2, Prkcb, Rac2, Ccl2, Cxcl16, Ccr5, Dock2, Cx3cl1, Vav1, Ptk2b, Cxcr4

**25 REACTOME CELL CYCLE CHECKPOINTS**	Hus1, Rpa1, Psma3, Psmd2, Psma2, Psmd9, Psmc6, Cdc6, Ube2d1, Psme1, Psmb2, Orc5l, Psmb4, Psmd13, Cdc16, Mcm5, Anapc4, Orc4l, Psma6, Rad17, Psmb6, Rfc5, Mcm3, Cdc27, Psmb3, Orc2l, Rfwd2, Trp53, Psme3, Orc3l, Psma1, Rad1, Atm, Psmd5, Mcm4, Mdm2, Rfc4, Psma4, Psmf1, Rfc2, Psmd8, Mcm2, Psma7, Psmd10, Chek2, Psmb10, Psmd14, Rfc3, Psme2, Bub1b, Ccne2, Rpa3, Psmb9, Psmb8, Mad2l1, Dbf4, Ube2c, Mcm7, Cdk1, Ccnb2

The top 25 Western diet gene sets whose member genes are predominantly upregulated with respect to the chronic control group. Column 1 includes the gene set name (bold) as provided by MSigDB and the rank of the gene set based on Normalized Enrichment Score (NES). Gene set names that are not in bold are gene sets that are redundant or partially redundant and included within larger bolded gene sets. The NES ranged from 2.97 (for KEGG Lysosome, the top ranked gene set) to 2.42 (REACTOME Cell Cycle Checkpoints, the 25th-ranked gene set). FDR $q < 1 \times 10^{-30}$. Column 2 includes those genes within the gene set that significantly contributed to the enrichment of the gene set. Genes within redundant gene sets are preceded by corresponding superscripts for the gene set to which they belong.

Supplemental Table 7 - Gene Set Enrichment Analysis. Negative enrichment: Western diet group vs. chronic control group

Gene Set	Genes Contributing to Core Enrichment
**1 G PROTEIN COUPLED RECEPTOR ACTIVITY**<br>6 RHODOPSIN LIKE RECEPTOR ACTIVITY<br>14 PEPTIDE RECEPTOR ACTIVITY	[6,14]Mas1, [6,14]Sstr4, [6,14]Grpr, [6]Adra2b, Gpr125, Grm7, [6]Tas2r107, [6]Tas2r121, [6,14]Sstr1, [6,14]Bdkrb2, [6]Adra2a, [6]Adra2c, Lphn3, [6]Tas2r137, Tmem11, [6]Htr5a, [6]Htr1f, [6,14]Sstr2, [6]Tas1r2, Celsr3, [6]Tas2r119, Pth2r, Bai1, [6,14]Oprm1, [6,14]Brs3, [6]Fshr, [6]Gpr139, [6]Chrm1, [6]Tas2r140, [6,14]Hcrtr2, [6]Pdgfrl, [6]Drd4, [6]Tas2r108, [6,14]Mchr1, [6]Htr2a, Gpr143, Grm2, [6]Olfr90, Gcgr, [6,14]Tacr2, [6]Tas2r130, [6,14]Oxtr, Grm3, Vipr2, Bai3, Grm5, [6,14]Sstr3, [6,14]Cckbr, [6,14]Mc3r, [6,14]Nmbr, [6,14]Galr3, Gpr123, [6]Mtnr1a, [6,14]Npbwr1, Gpr113, [6,14]Cckar, Gpr124, Gpr115, [6]Htr6, [6,14]Agtr2, [6]Pax8, Grm8, [6,14]Avpr1b, [6]Adra1d, [6]Htr1b, Lancl1, [6]Ghsr, [6]Olfr159, [6]Htr1a, Gpr128, [6]Htr1d, [6,14]Tacr1, [6]Tas2r118, [6]Trhr, [6]Mtnr1b, Glp2r, [6]Drd3, Gpr110, [6,14]Mc5r, Gabbr1, [6,14]Mc4r, [6]Tbxa2r, [6]Olfr15, [6]Olfr410, [6,14]Sstr5, [6]Tas1r3, Ghrhr, [6,14]Oprk1, [6,14]Gpr17, [6]Chrm5, [6,14]Kiss1r, [6]Gnrhr, [6,14]Galr2, [6,14]Nmur2, Lphn1, [6]Drd5, [6,14]F2rl3, [6,14]Avpr2
**2 APICAL JUNCTION COMPLEX**<br>**3 APICOLATERAL PLASMA MEMBRANE**	Cldn8, Cldn14, Cldn11, Cldn10a, Cldn4, Cldn17, Wnk4, Cldn6, Amotl1, Sorbs1, Pard3, Pard6a, Cldn1, Cldn18, Ash1l, Cldn3, Cldn12, Cldn16, Cldn23, Cldn19, Cldn7, Cldn20, Cldn9, Pdzd3, Cldn22
**4 NEUROTRANSMITTER BINDING**<br>5 NEUROPEPTIDE RECEPTOR ACTIVITY<br>7 NEUROTRANSMITTER RECEPTOR ACTIVITY<br>10 NEUROPEPTIDE BINDING	[7]Gabra2, [7]Chrna7, [7]Gabra6, [7]Gabra4, [7]Nmur1, [7]Chrm4, Mrgprx2, [5,7,10]Galr1, [7]Drd1a, [5,7,10]Tacr3, Adrb2, [7]Chrna6, [5,7,10]Sstr4, [5,7,10]Sstr1, [7]Chrna1, [5,7,10]Sstr2, [7]Chrnb4, [5,7,10]Brs3, [7]Gabrg2, [7]Chrm1, [5,7,10]Hcrtr2, [7]Drd4, [5,7,10]Mchr1, [5,7,10]Tacr2, [5,7,10]Sstr3, [5,7,10]Cckbr, [5,7,10]Nmbr, [5,7,10]Galr3, [5,7,10]Cckar, [7]Chrna5, [5,7,10]Tacr1, [7]Drd3, [5,7,10]Sstr5, [7]Chrne, [7]Chrm5, [5,7,10]Kiss1r, [5,7,10]Galr2, [5,7,10]Nmur2, [7]Drd5
**9 KEGG NEUROACTIVE LIGAND RECEPTOR INTERACTION**	Gipr, Chrna6, Npy2r, Mas1, Gabrg3, Sstr4, Grpr, Grid2, Taar9, Adra2b, Fshb, Grm7, Rxfp2, Sstr1, P2ry2, Bdkrb2 Adra2a, Adra2c, Gabrb2, Grik1, Grin2d, Ltb4r2, Chrna1, Htr5a Htr1f Taar2, Sstr2 Gabrb1, Fpr3, Grid1, Thra Pth2r, Htr2c, Oprm1 Chrnb4 P2ry4 Brs3, Fshr, Gabrg2, Chrm1, Hcrtr2, F2, Gpr50 Lhcgr, Drd4, Pard3, Mchr1, Htr2a, Grm2, Chrnb3, S1pr4, Taar6, Gpr83, Gcgr, Tacr2, S1pr5, Grin2b, Oxtr, Grm3, P2rx1, Vipr2, Taar5, Grm5, Ntsr2, Sstr3, Gabra1, Thrb, Cckbr, Mc3r, Nmbr, Galr3, Mtnr1a, Npbwr1, Cckar, Gzma, Htr6, Grin2c, Agtr2, Oprl1, Lpar2, Grm8, Avpr1b, Chrna5, Adra1d Htr1b, Ghsr Chrnd, Htr1a, Grik4, Grin3b, Gabre, Chrng, Calcr, Htr1d, Tacr1, Trhr, Mtnr1b, Glp2r, Drd3, Mc5r Gabrp, Gabbr1, Gria1, Mc4r, Tbxa2r, Grik5, Npffr1, Hrh4, Prss2, Hcrtr1, Sstr5, Ghrhr Npffr2 Oprk1, Chrne, Prlhr, Npy1r, Chrm5, Kiss1r, Gnrhr, Galr2, Gabrd, Nmur2, Lhb, Drd5, F2rl3, Avpr2, Prl, P2rx5
**11 REACTOME AMINE LIGAND BINDING RECEPTORS**<br>24 AMINE RECEPTOR ACTIVITY	Taar1, [24]Hrh3, [24]Chrm4, [24]Htr7, [24]Adrb1, [24]Adra1a, [24]Drd1a, [24]Hrh2, [24]Adrb2, Taar9, [24]Adra2b, Taar8c, [24]Adra2a, [24]Adra2c, [24]Htr5a, [24]Htr1f, Taar2, Htr2c, [24]Chrm1, [24]Drd4, [24]Htr2a, Taar6, Taar5, [24]Htr6, [24]Adra1d, [24]Htr1b, [24]Htr1a, [24]Htr1d, [24]Drd3, Hrh4, [24]Chrm5, [24]Drd5; (Htr3b – 24 ONLY)
**12 INTERCELLULAR JUNCTION**<br>8 TIGHT JUNCTION	[8]Cldn8, [8]Cldn14, [8]Cldn11, [8]Cldn10a, Pkp4, [8]Cldn4, [8]Cldn17, Vcl, Gjb1, [8]Wnk4, Dsc1, Bai1, [8]Cldn6, [8]Amotl1, Amtn, Sorbs1, [8]Pard3, Nrap, [8]Pard6a, [8]Cldn, [8]Cldn18, [8]Ash1l, [8]Cldn3, [8]Cldn12, [8]Cldn16, [8]Cldn23, Ctnna3, [8]Cldn19, Gjd3, Pdzd2, [8]Cldn7, [8]Cldn20, [8]Cldn9, Zyx, Pdzd3, [8]Cldn22
**13 STRUCTURAL CONSTITUENT OF MUSCLE**	Actn2, Actc1, Myl6b, 2900073G15Rik, Tcap, Myl6, Mybph, Mybpc3, Myh7, Myot, Asph, Myh11, Myl3, Mylpf, Smtn, Dmd, Tpm2, Myl2, Mybpc1, Mybpc2, Myom1

**15 CALCIUM CHANNEL ACTIVITY**	Cacna1e, Cacng5, Trpc5, Ryr1, Trpc1, Trpv6, Cacnb2, Cacnb1, Cacng1, Trpv5, Trpc4, Cacna1g, Cacng4, Cacna1c
**16 FEEDING BEHAVIOR**	Htr2c, Brs3, Hcrtr2, Npw, Mchr1, Pyy, Cckbr, Galr3, Cckar, Ghsr, Mc4r, Hcrtr1, Ghrl, Galr2, Nmur2
**18 VOLTAGE GATED CHANNEL ACTIVITY**<br>17 VOLTAGE GATED POTASSIUM CHANNEL ACTIVITY<br>20 VOLTAGE GATED CATION CHANNEL ACTIVITY	[17,20]Kcnh1, [20]Cacnb4, [17,20]Kcnj1, [20]Cacna1a, [17,20]Kcnq2, [17,20]Kcnc3, [20]Cacng2, [17,20]Kcns1, [17,20]Kcnh3, Clcn1, [20]Cacna2d1, [20]Nox1, [17,20]Kcnj12, [17,20]Kcnj6, [17,20]Kcne2, [17,20]Kcnb2, [20]Scn2a1, Clcnka, [20]Cacna1e, [20]Cacng5, [17,20]Kcnj10, [17,20]Kcnk1, [17,20]Kcnc1, [20]Scn11a, [17,20]Kcnq3, [17,20]Kcna5, [20]Cacnb2, [20]Cacnb1, [17,20]Kcns3, [20]Cacng1, Clcn2, [17,20]Kcnq5, [17,20]Kcnc4, [17,20]Kcna1, [17,20]Kcnj14, Clcnkb, [20]Cacna1g, [17,20]Kcna6, [20]Cacng4, [17,20]Kcnd3, [17,20]Kcnh2, [20]Cacna1c
**19 KEGG BASAL CELL CARCINOMA**	Fzd5, Wnt8b, Fzd2, Tcf7l2, Wnt11, Wnt1, Fzd9, Wnt10a, Fzd3, Wnt6, Wnt7b Fzd10, Fzd8, Wnt10b, Wnt5a, Shh, Dvl1, Smo, Wnt4, Wnt3a, Wnt8a, Wnt7a Tcf7l1, Wnt3, Dvl3, Ptch2, Gli1, Gli2, Stk36, Wnt2b, Hhip
**21 CONTRACTILE FIBER**	Tnni3, Ttn, Tpm3, Tnnt2, Des, Myl6b, 2900073G15Rik, Tnnc1, Vcl, Acta1, Cdk5r1, Svil, Myl3, Mylpf, Dmd, Tpm2, Mybpc1, Abra, Myom1
**22 ANION TRANSMEMBRANE TRANSPORTER ACTIVITY**	Slc20a1, Clcn1, Slc34a1, Slc4a2, Best1, Slc34a3, Slc17a2, Slc12a9, Fxyd1, Clca3, Clcnka, Slc5a5, Slc17a4, Abcc2, Slc12a3, Slc4a3, Slc22a8, Slc17a1, Slc12a4, Clcn2, Slc12a1, Abcc5, Slc34a2, Slc26a4, Slc17a3, Bsnd, Slc22a7, Slc13a4, Clcnkb, Nmur2
**23 REACTOME TIGHT JUNCTION INTERACTIONS**	Cldn8, Cldn14, Cldn11, Cldn10a, Crb3, Cldn4, Cldn17, Cldn6, Pard3, Pard6a, Cldn1, Cldn18, Cldn3, Cldn12, Cldn16, Cldn23, Cldn19, Cldn7, Cldn20, Cldn9, Cldn22
**25 DIGESTION**	Galr1, Slc15a1, Apoa4, Pgc, Sstr1, Ctrl, Akr1c21, Sstr2, Mep1b, Akr1d1, Pyy, Tff3, Cckbr, Npc1l1, Cckar, Tff2, Baat, Sst, Prss2, Galr2, Ppy, Ldlr

The top 25 Western diet gene sets whose member genes are predominantly downregulated with respect to the chronic control group. Column 1 includes the gene set name (bold) as provided by MSigDB and the rank of the gene set based on Normalized Enrichment Score (NES). Gene set names that are not in bold are gene sets that are redundant or partially redundant and included within larger bolded gene sets. The NES ranged from -3.43 (for G Protein Coupled Receptor Activity, the top ranked gene set) to -1.92 (Digestion, the 25th-ranked gene set). FDR q range: 0 – 0.015. Column 2 includes those genes within the gene set that significantly contributed to the enrichment of the gene set. Genes within redundant gene sets are preceded by corresponding superscripts for the gene set to which they belong.
